# Comparative Physiological and Transcriptome Analysis Reveal the Molecular Mechanism of Melatonin in Regulating Salt Tolerance in Alfalfa (*Medicago sativa* L.)

**DOI:** 10.3389/fpls.2022.919177

**Published:** 2022-07-13

**Authors:** Shuxia Li, Yuan Wang, Xueqin Gao, Jian Lan, Bingzhe Fu

**Affiliations:** ^1^School of Agriculture, Ningxia University, Yinchuan, China; ^2^Ningxia Grassland and Animal Husbandry Engineering Technology Research Center, Yinchuan, China

**Keywords:** melatonin, salt stress, transcriptome, plant hormone, signal transduction, *Medicago sativa*

## Abstract

As a high-quality legume forage, alfalfa is restricted by various abiotic stresses during its growth and development. Melatonin is a multifunctional signaling molecule that involves in plant defense against multiple stresses. However, little is known about its downstream signaling pathway and regulatory mechanisms in salt stress of alfalfa. In this study, we investigated the protective effects and key regulatory pathways of melatonin on alfalfa under salt tolerance. The results showed that melatonin promoted the growth of alfalfa seedlings under salt stress, as demonstrated by higher plant height, leaf area, and fresh weight. Melatonin treatment resulted in an increase in the photosynthetic capacity and starch content of alfalfa. Moreover, melatonin decreased cell membrane damage and reactive oxygen species (ROS) accumulation by enhancing antioxidant defense activity under salt stress conditions. Transcriptome sequencing (RNA-seq) analysis revealed that melatonin mainly induced the transcription of genes involved in Ca^2+^ signaling (cyclic nucleotide gated channel, *CNGCs*; cam modulin/calmodulin-like protein, *CAM/CMLs* and calcium-dependent protein kinase, *CDPKs*), starch and sucrose metabolism (α-amylase, *AMYs*; β-amylase, *BAMs*; starch synthase, *SSs* and sucrose synthase, *SUSs*), plant hormone signal transduction (auxin/indole acetic acid protein, *AUX*/*IAAs*; ABA receptor, *PYL4*; protein phosphatase 2C, *PP2Cs*; scarecrow-like protein, *SCLs* and ethylene-responsive transcription factor 1B, *ERF1B*), and key transcription factors (*C3Hs, MYBs, ERFs*, and *WRKYs*). Specifically, we focused on starch and sucrose metabolism and plant hormone signal transduction pathways. The interactions between melatonin and other phytohormones occurred *via* regulation of the expression of genes involved in hormone signaling pathways. In addition, melatonin increased the contents of endogenous melatonin, auxin, gibberellic acid (GA_3_), salicylic acid, brassinosteroids, and ethylene, while decreasing the abscisic acid content under salt stress. In summary, this study established a regulatory network for melatonin-induced key signaling pathways and functional genes under salt stress and provided a theoretical basis for salt tolerance breeding in alfalfa.

## Introduction

Soil salinization is a severely adverse environmental factor that threatens agricultural sustainability and global food security (Cheeseman, 2016). Salinization limits plant growth, development, productivity and quality, especially in arid, and semiarid regions (Pang et al., 2016). Unfortunately, salt stress influences more than 800 Mha of all irrigated lands worldwide, and this problem continues to worsen (Yang and Guo, 2018; FAO, 2019). The excessive salts in the soil solution cause osmotic, ionic, and secondary stresses on plants. Salt stress is commonly caused by high concentrations of sodium ions (Na^+^) and chloride ions (Cl^−^) in soil (Ismail and Horie, 2017). Plants have evolved diverse mechanisms to cope with salt stress. Currently, several regulatory components have been found to play important roles in signal transduction under salt stress, including ion balance regulation, reactive oxygen species (ROS) homeostasis modulation, and plant hormone metabolism (Horvath et al., 2015).

Melatonin (N-acetyl-5-methoxytryptamine) is a highly conserved and ubiquitous indoleamine molecule in the plant kingdom (Paredes et al., 2009). Numerous studies have revealed that melatonin, as a growth regulator or defense response biostimulator, is involved in regulating various biological processes in plants, including seed germination, seedling growth, root morphology, leaf senescence, nutrient absorption, floral transition, fruit ripening, as well as multiple abiotic and biotic stress responses (Fan et al., 2018; Arnao and Hernandez-Ruiz, 2019; Sun et al., 2021). The biosynthesis of melatonin in higher plants begins with tryptophan and is catalyzed by the following four successive enzyme reactions: Tryptophan decarboxylase (TDC), tryptamine 5-hydroxylase (T5H), serotonin N-acetyltransferase (SNAT), and N-acetylserotonin methyltransferase (ASMT)/caffeic acid O-methyltransferase (COMT) (Back et al., 2016).

Melatonin is a powerful antioxidant and free radical scavenger that suppresses peroxidative metabolism in plants under abiotic stresses (Arnao and Hernandez-Ruiz, 2019; Sharma et al., 2020). Experimental evidence proves that melatonin can exert its antioxidant capacity by scavenging excessive ROS and reactive nitrogen species (RNS) directly (Martinez et al., 2018). Melatonin increases a wide spectrum of plant stress tolerance indirectly by enhancing the activities of antioxidant enzymes (SOD, CAT, POD and GPX, etc.), improving photosynthesis and redox homeostasis, activating downstream signals and regulating the expression of stress-responsive genes (Zhang and Zhang, 2014; Liu et al., 2020). Melatonin interacts with various phytohormones such as auxin (IAA), ethylene (ETH), jasmonic acid (JA), salicylic acid (SA), abscisic acid (ABA), and brassinosteroids (BRs) to participate in stress responses (Arnao and Hernandez-Ruiz, 2019). Melatonin promotes ethylene biosynthesis by regulating the expression of *ACS1* gene; thus, enhancing the salt tolerance of grapevines (Xu et al., 2019). In the recent years, there has been much research on melatonin in salt stress and in a variety of plant species (Wei et al., 2015; Yan et al., 2019; Liu et al., 2020). Nevertheless, the signaling network of melatonin-mediated salt stress responses in plants is complex and remains largely obscure, especially in forages.

Alfalfa (*Medicago sativa* L.) is an important perennial legume species that is widely cultivated around the world. The planting area of alfalfa is approximately 32.2 Mha worldwide, 11% (~3.77 Mha) of which are planted in China (Shi et al., 2017). Alfalfa provides high protein content and excellent palatability of forage for animals and improves soil fertility. It is therefore regarded as the “king of forages.” However, alfalfa quality and yield are severely constrained by adverse environmental factors, such as soil salinity and limited water supplies in agriculture (Singer et al., 2018). Therefore, combining physiological, biochemical, and molecular approaches to improve the salt tolerance of alfalfa is significant for the production of high-quality alfalfa on saline-alkali land.

In this study, we investigated the physiological and molecular mechanisms of melatonin-mediated salt stress tolerance in alfalfa plants. The results reveal that melatonin could improve alfalfa salt tolerance by enhancing photosynthetic capacity and the antioxidant defense system and reducing membrane damage and ROS accumulation at the physiological level. Based on transcriptome data, we focused on the key genes involved in starch and sucrose metabolism and hormone signaling pathways. The results of this study aid in understanding the molecular mechanism underlying melatonin-mediated salt stress tolerance in alfalfa.

## Materials and Methods

### Plant Materials and Treatments

Alfalfa (*Medicago sativa* L. cv. zhongmu 1#) seeds were surface-sterilized with 75% (v/v) ethanol and 5% sodium hypochlorite solution and then germinated on wet filter paper in Petri dishes for 6 days at 25°C. For the hydroponic experiment, seedlings with uniform growth were transferred into plastic containers filled with Hoagland solution in a growth chamber (25°C, 16-h light/8-h dark cycle, 60% relative humidity). For soil culture, the seedlings were transplanted into plastic pots that were 7 cm in diameter and 8.5 cm in depth with vermiculite and watered with Hoagland nutrient solution. After 3 weeks of incubation, the plants with uniform growth were treated with the following different solutions: (i) Control (CK), Hoagland nutrient solution alone; (ii) salt stress (S), Hoagland nutrient solution with 150-mM NaCl; (iii) melatonin (M), Hoagland nutrient solution plus melatonin; and (iv) salt stress with melatonin (SM), Hoagland nutrient solution with 150-mM NaCl plus melatonin. The NaCl treatment concentration was set according to the literatures (Benabderrahim et al., 2020; Yu et al., 2021). Each treatment contained 36 pots with one plant per pot. Alfalfa plants were treated with 10-μM melatonin in hydroponic experiments to observe the root phenotype. In soil culture pre-experiments, four different concentrations (0, 20, 50, and 100 μM) of melatonin were applied to choose the appropriate concentration of melatonin treatment. After treatment, the leaf samples were subsequently frozen in liquid nitrogen and stored at −80°C. The experiment was repeated 3 times.

### Determination of Physiological Parameters and Hormone Contents

According to the growth phenotype of alfalfa, the representative individuals from each group were photographed on the 15th day of salt treatment, and the plant height, shoot fresh weight, and leaf area were measured. The net photosynthetic rate (Pn) of the third fully expanded leaf was determined using a Li-6400 portable photosynthesis system (Lincoln, NE, USA) according to the manufacturer's instructions. The photosynthetic photon flux density (PPFD) and the external CO_2_ concentration were set at 1,000 μmol m^−2^ s^−1^ and 400 μmol mol^−1^, respectively. Electrolyte leakage was detected by conductometer according to the methods of a previous study (Li et al., 2019a). The malondialdehyde (MDA) content was quantified by a thiobarbituric acid method (Puckette et al., 2007). The content of hydrogen peroxide (H_2_O_2_) and superoxide anion radical (O${}_{2}^{\cdot -}$) were spectrophotometrically determined as described by Jiang and Zhang (2001). The activities of superoxide dismutase (SOD), peroxidase (POD), glutathione S-transferase (GST), ascorbate peroxidase (APX), as well as the levels of proline, total soluble sugar, and total starch were determined using the corresponding plant kits (Solarbio Science & Technology Co., Ltd. Beijing, China) according to the manufacturer's instructions.

The Na^+^ and K^+^ contents were measured following our previous work (Li et al., 2019b). Approximately 20–50 mg of dry powder sample from each treatment was weighed and dissolved with acetic acid solution. Then, the Na^+^ and K^+^ contents of the extract were determined using a flame photometer.

The content of endogenous hormones, including melatonin, ABA, IAA, gibberellic acid (GA_3_), SA, BR, and ETH, in the alfalfa leaf samples were measured using HPLC–MS/MS analysis as described in the previous studies, with some modifications (Liu et al., 2018; Zhang et al., 2018).

### The RNA Sequencing and Bioinformatics Analysis

The RNA samples for the transcriptome were extracted from the leaves of CK-, S-, M-, and SM-treated alfalfa plants. Each treatment was represented by three biological replicates of leaf samples. The total RNA was isolated and purified using TRIzol reagent (Invitrogen, Carlsbad, CA, USA) following the manufacturer's procedure. The RNA amount and purity of each sample were quantified using a NanoDrop ND-1000 (NanoDrop, Wilmington, DE, USA). The integrity of RNA was assessed by a Bioanalyzer 2100 (Agilent, CA, USA) with RIN > 7.0 and confirmed by electrophoresis with denaturing agarose gel. Sequencing library construction and sequencing were performed using Illumina Novaseq™ 6000 (LC-Bio Technology CO., Ltd., Hangzhou, China) following the vendor's recommended protocol. The adaptor contamination reads were removed using Cutadapt software (version 1.9), and the low-quality bases and undetermined bases were removed from the raw data (Wang et al., 2012). HISAT2 software (version: 2.0.4) was used to map clean reads to the genome (Kim et al., 2015).

The mapped reads of each sample were assembled using StringTie with the default parameters. Then, all transcriptomes from all samples were merged to reconstruct a comprehensive transcriptome using gffcompare software. StringTie and Ballgown software were used to estimate the expression levels of all transcripts and perform gene expression level analysis by calculating the fragments per kilobase of transcripts per million (FPKM) mapped reads. Genes with fold change more than 2 or < 0.5 and *p* < 0.05 were defined as differentially expressed. The differential expression analysis was conducted using the DESeq2 R package (Pertea et al., 2015). The Gene Ontology (GO) terms and Kyoto Encyclopedia of Genes and Genomes (KEGG) pathway enrichment analysis of differentially expressed genes (DEGs) were conducted using the R package (Minoru et al., 2008; Young et al., 2010).

### Quantitative Real-Time PCR (qRT-PCR) Validation

Total RNA was isolated and purified using TRIzol reagent (Invitrogen, Carlsbad, CA, USA). The RNA samples were reverse-transcribed with HiScript II Q RT SuperMix for qPCR (+gDNA wiper) (Vazyme, Nanjing, China) according to the manufacturer's protocol. Twenty genes were randomly selected for the qRT-PCR assay to validate the RNA-seq data. The qRT-PCR was performed using SYBR Premix on a Bio-Rad CFX 96 RT-PCR System (Bio-Rad, Inc., CA, USA). Three independent biological replicates and three replicate reactions for each sample were performed in qRT-PCR assays. The alfalfa β*-actin* gene was used as an internal control for expression analysis (Long et al., 2014). The relative expression levels of selected genes were calculated by the 2^−Δ*ΔCt*^ method (Livak and Schmittgen, 2001). The gene-specific primer pairs used in qRT-PCR are listed in Supplementary Table 1.

### Statistical Analysis

Statistical analysis was performed using SPSS 20.0 statistical software (SPSS Inc., Chicago, IL, USA). Significant differences in the physiological parameters were analyzed by one-way ANOVA followed by Duncan's test. The differences between individual means were considered significant at *p* < 0.05.

## Results

### Detection of the Alleviating Effects of Exogenous Melatonin on Salt Stress in Alfalfa

**Figure 1:**
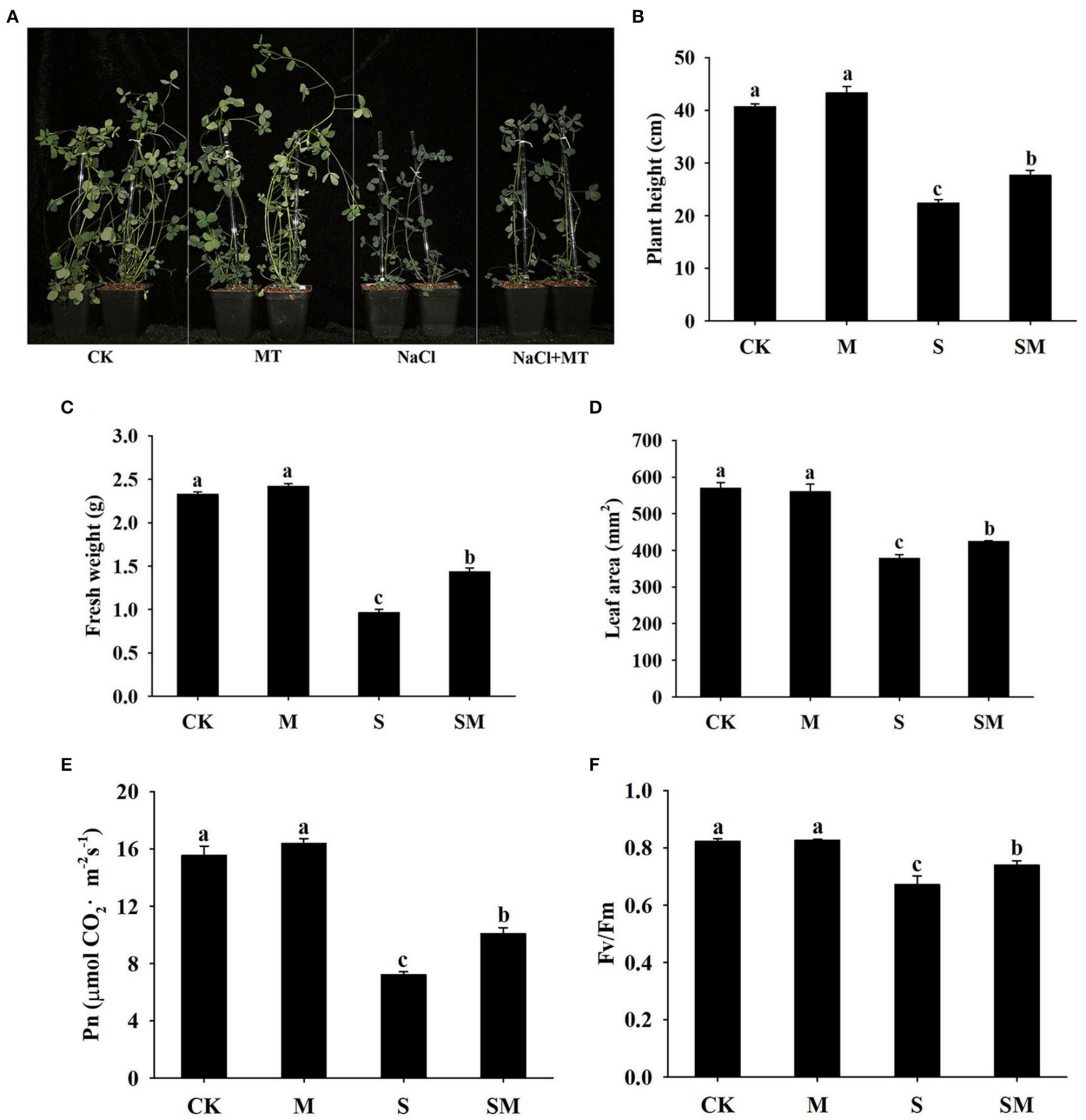
Effect of exogenous melatonin on the phenotype traits and photosynthetic capacity of alfalfa seedlings after 15 day of 150-mM NaCl treatment. **(A)** The phenotype of a representative individual from each treatment. **(B)** Plant height. **(C)** Fresh weight. **(D)** Leaf area. **(E)** Net photosynthetic rate (Pn). **(F)** Fv/Fm. The data are means ± SE (*n* = 6) and different letters are significantly different (*p* < 0.05).

**Figure 2:**
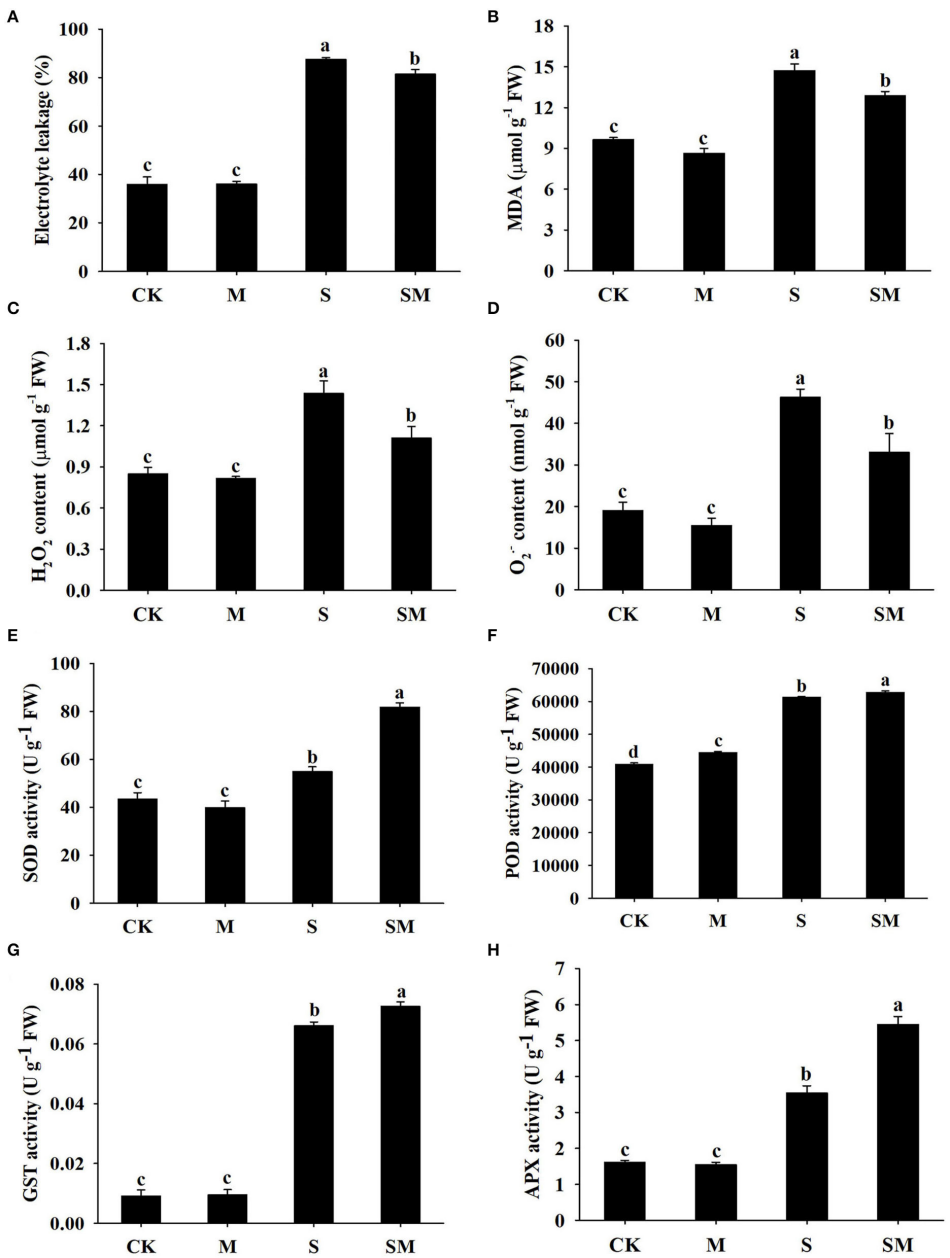
Effect of exogenous melatonin on **(A)** electrolyte leakage, **(B)** MDA content, **(C)** H_2_O_2_ content, **(D)** O${}_{2}^{.-}$ content, **(E)** SOD, **(F)** POD, **(G)** GST, and **(H)** APX of alfalfa seedlings after 15 days of 150-mM NaCl treatment. Data are means ± SE (*n* = 3) and different letters are significantly different (*p* < 0.05).

To evaluate whether melatonin can improve the salt stress tolerance of alfalfa, the seedlings were subjected to NaCl and melatonin treatment, and the phenotypic traits were observed (Figure 1A and Supplementary Figures 1A,D). Compared with the control plants, 10-μM melatonin treatment significantly increased the root length and root dry weight of alfalfa in hydroponic experiments. Salt stress inhibited the root length and root dry weight, while the application of melatonin significantly improved these parameters (Supplementary Figures 1B,C). In the soil culture pre-experiments, the plant height and Pn of the plants treated with 50-μM melatonin were higher than those of other treatments (0, 20 and 100 μM) under salt stress (Supplementary Figures 1E,F). Therefore, we selected 50-μM as the optimal concentration for melatonin treatment in subsequent experiments. Compared with the control, salt stress obviously inhibited the growth of alfalfa seedlings, as evidenced by the declines in plant height, leaf area, fresh weight, and photosynthetic parameters: Pn and Fv/Fm; however, the application of melatonin dramatically mitigated this effect in the soil culture experiments (Figures 1B–F). In addition, melatonin treatment significantly decreased the electrolyte leakage and MDA content under salt stress, but had no obvious effect on control plants (Figures 2A,B). Salt treatment resulted in a significant increase in H_2_O_2_ and O${}_{2}^{.-}$ contents, while the melatonin treatment reduced them by 0.8 and 0.7 times, respectively (Figures 2C,D). Plants have involved a complex antioxidant defense system to cope with abiotic stress-triggered oxidative damage, including some functionally correlated antioxidant enzymes. As shown in Figures 2E–H, salt stress significantly increased the SOD, POD, APX, and GST enzymatic activities in comparison with control plants. However, the activity of these enzymes was remarkably higher in melatonin-treated plants than in non-treated plants under salt stress.

**Figure 3:**
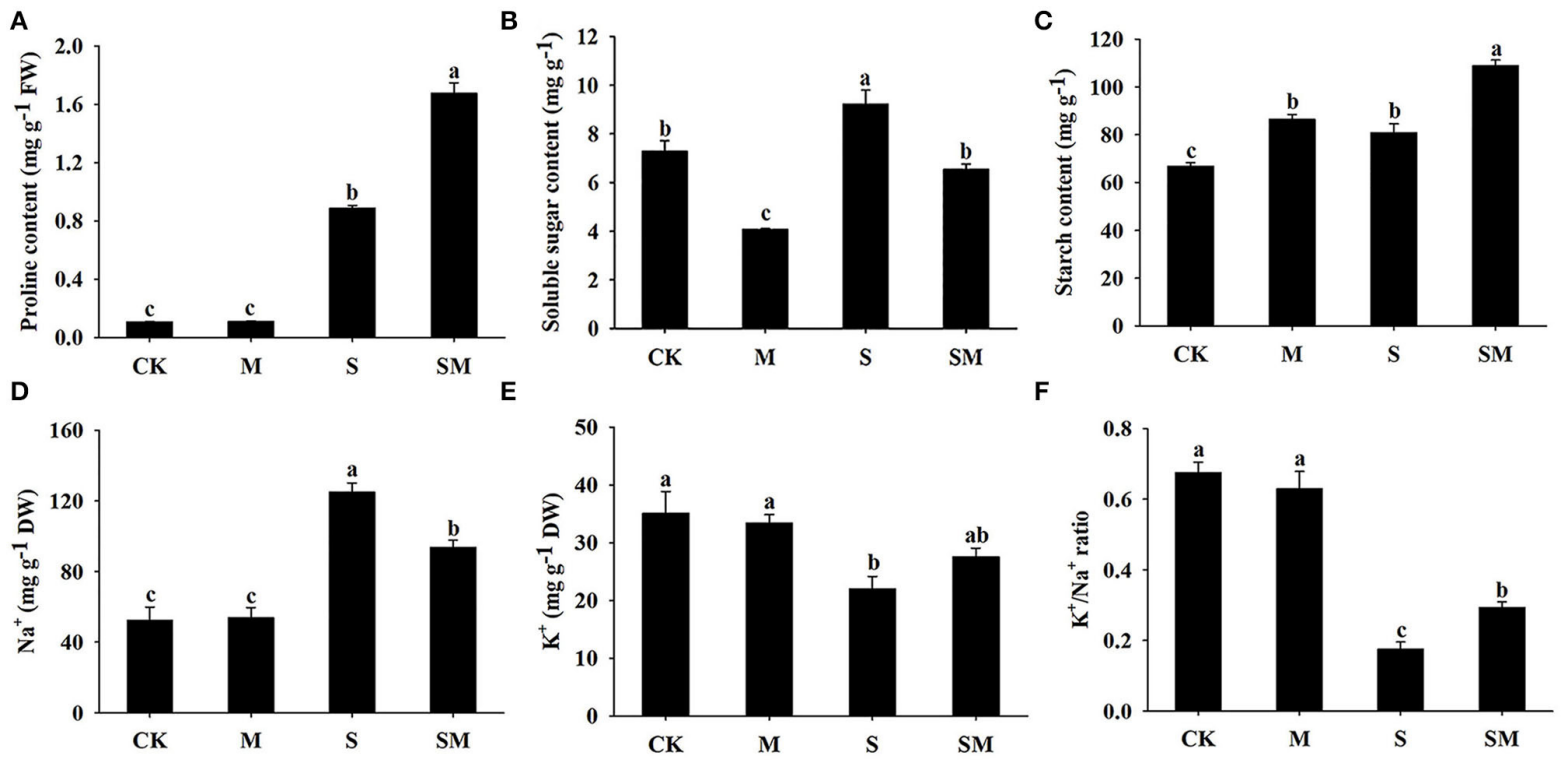
Effect of exogenous melatonin on the contents of **(A)** proline, **(B)** soluble sugar, **(C)** starch, **(D)** Na^+^, **(E)** K^+^, and **(F)** K^+^/Na^+^ ratio of alfalfa seedlings after 15 days of 150-mM NaCl treatment. The data are means ± SE (*n* = 3) and different letters are significantly different (*p* < 0.05).

Melatonin had no significant effect on the proline content under control conditions. When salt stress was applied, the proline content was significantly increased, while the melatonin-treated plants exhibited a higher level of proline accumulation (Figure 3A). The soluble sugar content of the seedlings was greatly increased under salt stress, while melatonin treatment decreased the soluble sugar content in both the control and salt-treated plants by 44.08 and 28.81%, respectively (Figure 3B). As shown in Figure 3C, melatonin treatment increased the starch content in alfalfa plants before and after salt stress. Salt stress resulted in significantly higher starch level than that in the control plants. The K^+^/Na^+^ ratio, a key indicator of salt stress mitigation, was significantly decreased under salt stress conditions due to elevated Na^+^ and reduced K^+^. However, melatonin treatment markedly increased the salt-induced K^+^/Na^+^ ratio in alfalfa (Figures 3D–F).

### Transcriptome Sequencing (RNA-Seq) and DEGs Analysis

To further elucidate the molecular mechanism underlying melatonin-induced salt stress tolerance in alfalfa, transcriptome analyses of alfalfa leaves were performed on 12 samples (CK-1/-2/-3, M-1/-2/-3, S-1/-2/-3, and SM-1/-2/-3) using the Illumina Novaseq™ 6000 sequencing platform. A total of 92.78 GB raw reads were obtained from all tested samples. More than 6.34 GB average clean data were obtained for each RNA-seq sample, and the valid data ratio was above 92% (Q20 > 99.94% and Q30 > 98.60%) (Supplementary Table 2). The valid data were mapped to the reference genome by HISAT software, and over 90.40% mapped reads were obtained. A total of 164,632 transcripts were generated in the alfalfa transcriptome data, and 126,412 and 49,751 transcripts were annotated in GO and KEGG, respectively (Supplementary Table 3). Three independent biological replicates of each treatment were clustered in a PCA, and the repeatability within each group (CK, M, S, and SM) and the discrimination between groups were good (Supplementary Figure 2). The transcriptome data were reliable enough to support further analysis.

**Figure 4:**
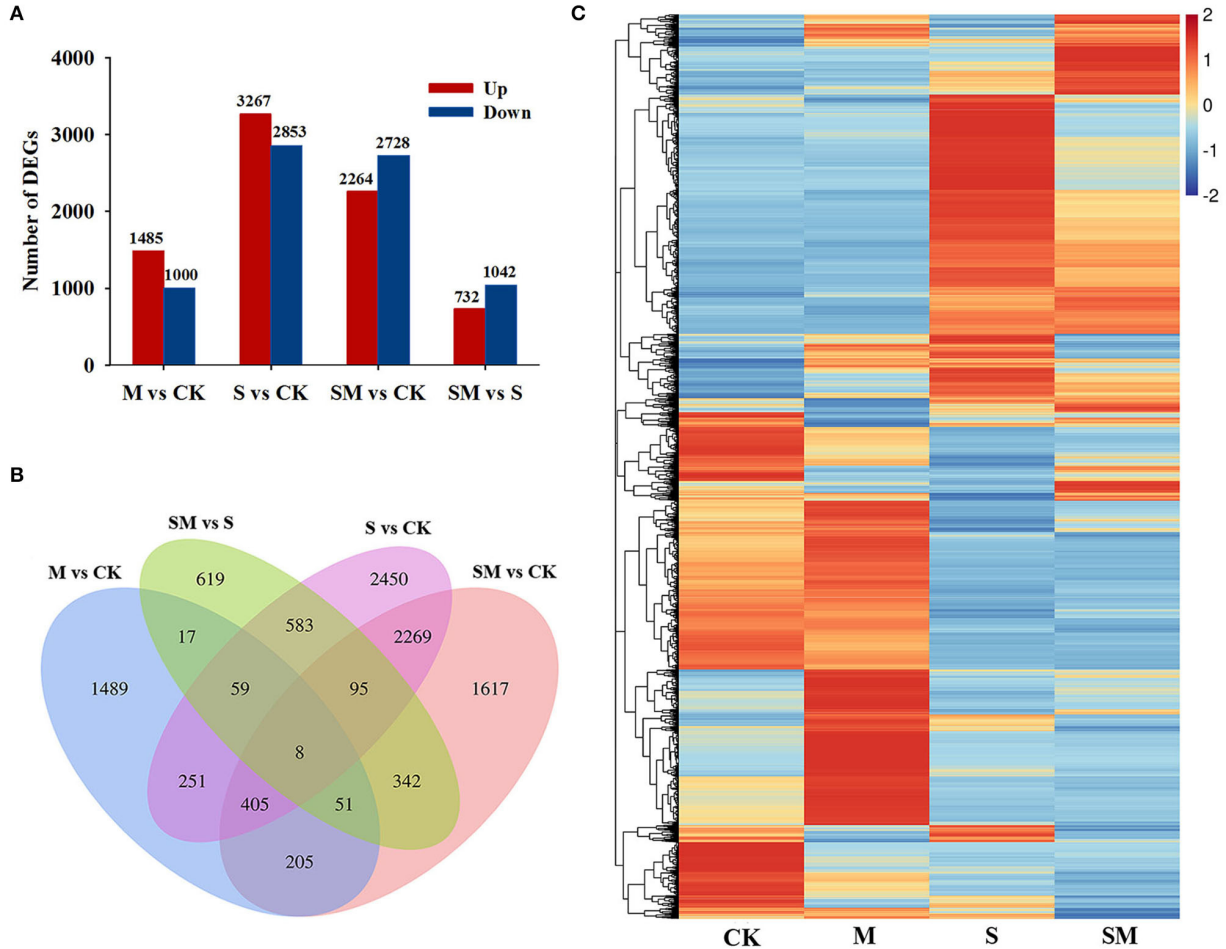
Transcriptional profiles of alfalfa seedlings under different treatments. **(A)** Numbers of differentially expressed genes (DEGs) in the transcriptome data. **(B)** Venn diagram showing numbers of overlapping DEGs in the transcriptome data. **(C)** Hierarchical clustering (HCL) analysis of the DEGs under different treatments.

Compared with the control plants, 2,485 (1,485 upregulated and 1,000 downregulated genes), 6,120 (3,267 upregulated genes and 2,853 downregulated genes) and 4,992 (2,264 upregulated and 2,728 downregulated genes) DEGs were identified in the “M *vs*. CK,” “S *vs*. CK,” and “SM *vs*. CK” comparisons, respectively. A total of 1,774 DEGs, including 732 upregulated and 1,042 downregulated genes, were obtained in “SM *vs*. S” (Figure 4A and Supplementary Table 4). A Venn diagram showed that 723 DEGs were affected by both melatonin and salt stress (the intersection of “M *vs*. CK” and “S *vs*. CK”). Melatonin affected 135 DEGs under both the control and salt stress conditions (the intersection of “M *vs*. CK” and ”SM *vs*. S”). Additionally, 67 DEGs were found in the intersection of “M *vs*. CK,” “S *vs*. CK,” and “SM *vs*. S” (Figure 4B), indicating that these genes may be involved in the melatonin-induced salt stress response. To determine the expression patterns of DEGs under different experimental conditions, the FPKM values of the DEGs were used for hierarchical cluster analysis (HCL) (Figure 4C).

### Gene Ontology Enrichment Analysis of the DEGs

To further clarify the functional categories of the DEGs induced by melatonin treatment, GO enrichment analysis was performed. The DEGs from “M *vs*. CK,” “S *vs*. CK,” “SM *vs*. CK,” and “SM *vs*. S” were assigned to 2,119, 2,958, 2,799, and 1,906 GO terms, respectively (Supplementary Table 5). As shown in Supplementary Figure 3, the identified DEGs were classified into the following three major GO categories: Biological process, cellular component, and molecular function. The top-25, top-15, and top-10 GO terms of biological process, cellular component and molecular function were selected for display and analysis according to the number of DEGs. The GO terms in the biological process category were mainly related to biological process, regulation of transcription, DNA-templated, transcription, DNA-templated, oxidation–reduction process, defense response, and protein phosphorylation. In the cellular component category, nucleus, plasma membrane, cytoplasm, integral component of membrane, chloroplast and cytosol were the main GO terms. In molecular function, the top enriched GO categories were involved in molecular function, protein binding, ATP binding, DNA binding transcription factor activity, metal ion binding and DNA binding. The GO terms in the three major categories were different among the comparison groups, which indicates that the salt resistance processes regulated by melatonin in alfalfa are complex.

### Kyoto Encyclopedia of Genes and Genomes Pathway Enrichment Analysis of DEGs

**Figure 5:**
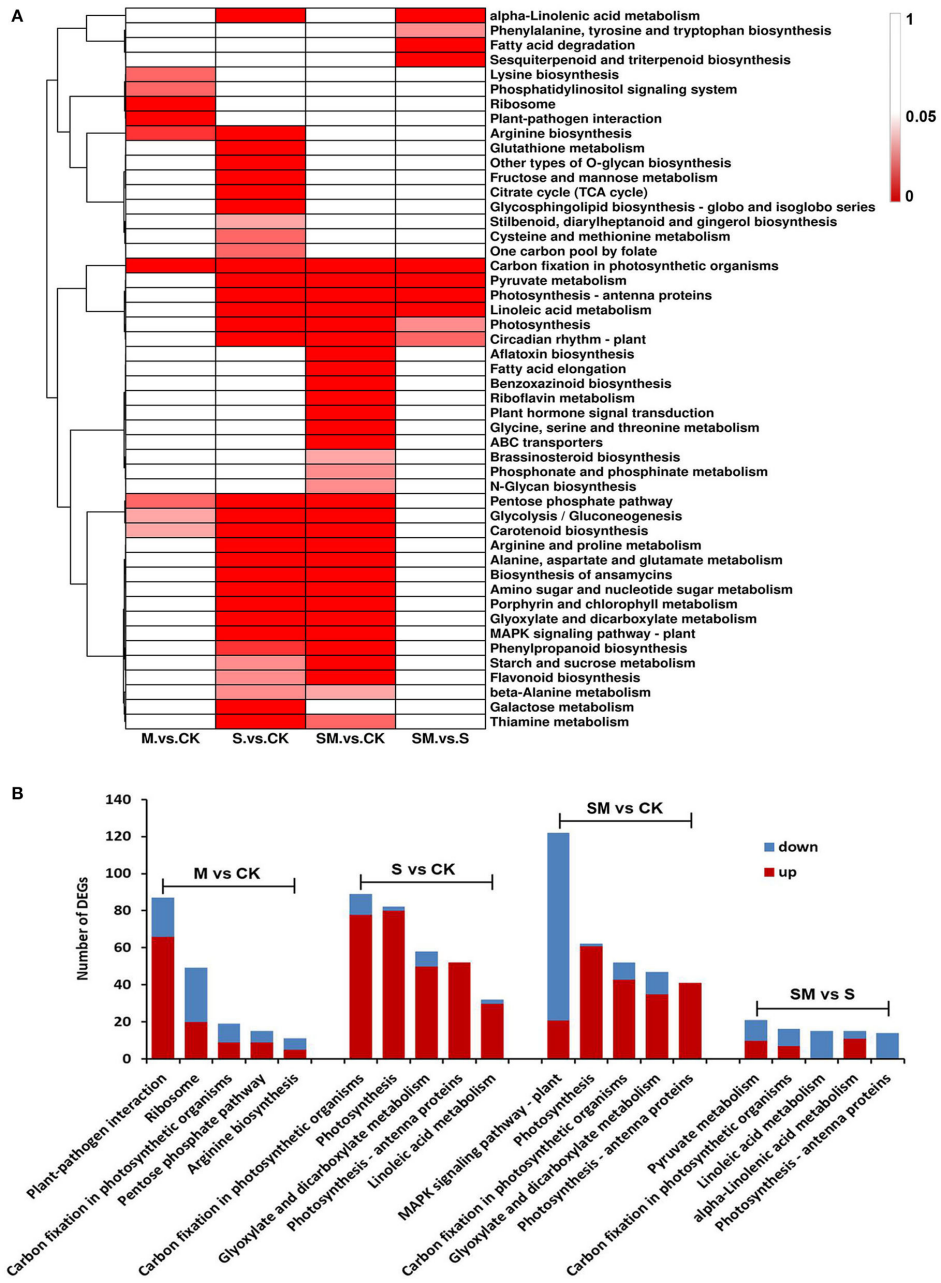
KEGG pathway enrichment analysis of DEGs in different comparisons. **(A)** Heat map analysis of the significant *p*-values (*p* < 0.05) of KEGG term in different comparisons. **(B)** Top-5 KEGG pathways of DEGs in different comparisons.

Genes usually interact with each other to play roles in certain biological functions. To further understand the biological functions of genes involved in the melatonin-regulated salt resistance process, the enriched KEGG pathways were identified using the KEGG database (Supplementary Table 6). In total, 49 KEGG pathways were significantly enriched in at least one comparison (*p* < 0.05) (Figure 5A). A total of 93,232 and 10 KEGG pathways were significantly enriched in the “M *vs*. CK,” “S *vs*. CK,” “SM *vs*. CK” and “SM *vs*. S” comparisons, respectively. As shown in Figure 5A, aflatoxin biosynthesis, fatty acid elongation, benzoxazinoid biosynthesis, riboflavin metabolism, plant hormone signal transduction, glycine, serine and threonine metabolism, ABC transporters, brassinosteroid biosynthesis, phosphonate and phosphinate metabolism and N-glycan biosynthesis were the only significantly enriched metabolic pathways in “SM *vs*. CK.” The pentose phosphate pathway, glycolysis/gluconeogenesis and carotenoid biosynthesis pathways were significantly enriched in the “M *vs*. CK,” “S *vs*. CK” and “SM *vs*. CK” comparisons. Furthermore, carbon fixation in photosynthetic organism pathway was significantly changed in all comparisons and enriched in the top five (Figure 5B). Analysis of the top five KEGG pathway showed that the DEGs in the “S *vs*. CK” and “SM *vs*. CK” comparisons were enriched in photosynthesis and glyoxylate and dicarboxylate metabolism. The DEGs involved in photosynthesis–antenna proteins were observed in the “S *vs*. CK,” “SM *vs*. CK” and “SM *vs*. S” comparisons. In addition, linoleic acid metabolism was enriched in “S *vs*. CK” and “SM *vs*. S” (Figure 5B). The results indicate that highly enriched pathways may be essential for melatonin-regulated salt resistance in alfalfa.

### Detection of key Melatonin-Induced Genes and Transcription Factors (TFs) Under Salt Stress

**Figure 6:**
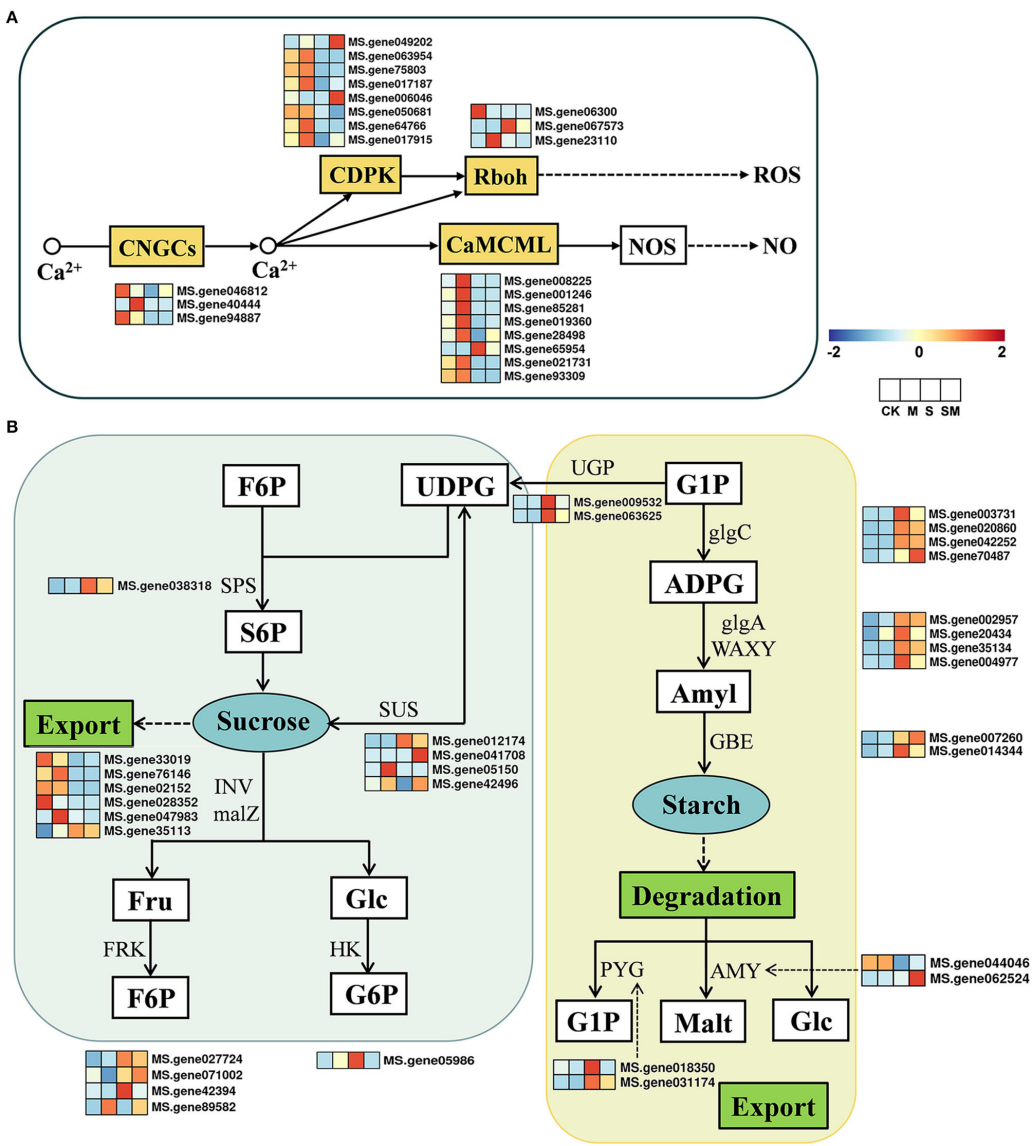
Expression profiles of the key genes involved in **(A)** Ca^2+^ signal pathway and **(B)** starch and sugar metabolism in response to MT and salt treatment.

Plants can trigger multiple biological processes to regulate gene transcription and physiological adaptation under unfavorable conditions (Deng et al., 2020). We examined the profiles of the genes involved in the plant-pathogen interaction pathway and starch and sucrose metabolism (Figure 6). In the plant–pathogen interaction pathway, a total of 87 DEGs were identified in “M *vs*. CK,” including 66 upregulated and 21 downregulated DEGs. Under salt stress, most of the genes involved in Ca^2+^ signal transduction and WRKY TFs were downregulated in “S *vs*. CK” and “SM *vs*. CK,” while the opposite was true in “M *vs*. CK” and “SM *vs*. S” (Supplementary Table 7). The expression profiles of 22 DEGs involved in the Ca^2+^ signaling pathway are shown in Figure 6A. Melatonin induced the expression of genes encoding cyclic nucleotide-gated ion channel (*CNGC20*), calmodulin/calmodulin-like protein (*CML7, CML11, CML24, CML45*, and *CML48*), calcium-dependent protein kinase (*CDPK1*), and respiratory burst oxidase (*RbohB*) in “M *vs*. CK” (Supplementary Table 7).

The expression profiles of 32 DEGs involved in starch and sucrose metabolism are shown in Figure 6B. According to our results (Supplementary Table 8), 35 genes were differentially expressed in “M *vs*. CK,” with 24 upregulated and 11 downregulated genes, while 25 DEGs were identified in “SM *vs*. S.” In “M *vs*. CK,” sucrose synthase (*SUSs*), glycoside hydrolase family 17 (*GH17*), and β-amylase 3 (*BAM3*) were downregulated, while trehalose-phosphate phosphatase (*TPP*), endoglucanase 6 (*EDGL6*), and glucan endo-1,3-beta-glucosidase 13 (*EGLC13*) were upregulated. Specifically, the gene encoding fructokinase (*FRK7*) was upregulated in both “M *vs*. CK” and “SM *vs*. S,” while *EGLC14* was upregulated in “M *vs*. CK” and downregulated in “SM *vs*. CK.” In response to salt stress, 63 DEGs (31 upregulated and 32 downregulated genes) were identified in both “S *vs*. CK” and “SM *vs*. CK.” The genes encoding *EGLCs, TPP*, hexokinase 3 (*HXK3*), *SUSs*, α-amylase (*AMY*), beta-glucosidase 24 (*BGLU24*), and *FRK4* were downregulated, whereas *BAM*, starch synthase (*SSs*), *BGLUs*, sucrose-phosphate synthase 2 (*SPS2*), granule-bound starch synthase (*GBSSs*), and *AMY2* were upregulated under salt stress.

**Figure 7:**
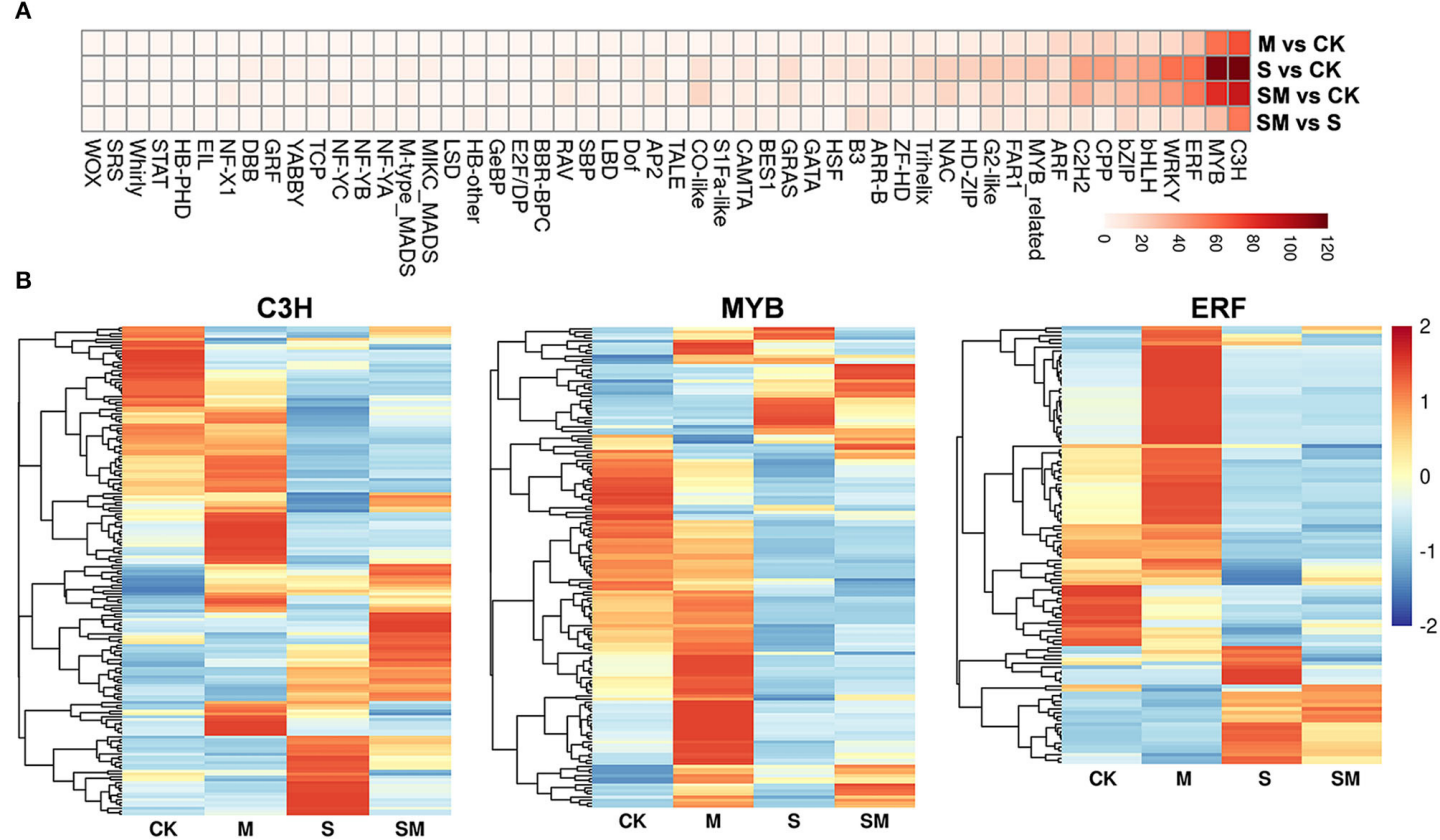
Expression analysis of TF families in different comparisons. **(A)** Heatmap analysis of TF families. **(B)** The differential expression pattern of MYB, NAC, and WRKY families.

Transcription factors play a critical role in regulating upstream stress signal transduction to downstream gene transcription and specific biological processes. A total of 2,111 TFs from 52 families showing differential expression were identified (Figure 7A and Supplementary Table 9). The C3H, MYB, and ERF families were the three most abundant TF families in each comparison. The expression profiles of the three TF families in four comparisons are presented in Figure 7B. We found 373 and 278 TFs were differentially expressed in “M *vs*. CK” and “SM *vs*. S”; the largest proportion of the C3H family contained 69 and 55 DEGs, respectively. Interestingly, 147 TFs were differentially expressed in “SM *vs*. S,” but not in “S *vs*. CK,” which suggests that these TFs are participated in salt resistance regulated by melatonin.

### Detection of Melatonin-Induced Genes Involved in Phytohormone Signal Transduction Under Salt Stress

**Figure 8:**
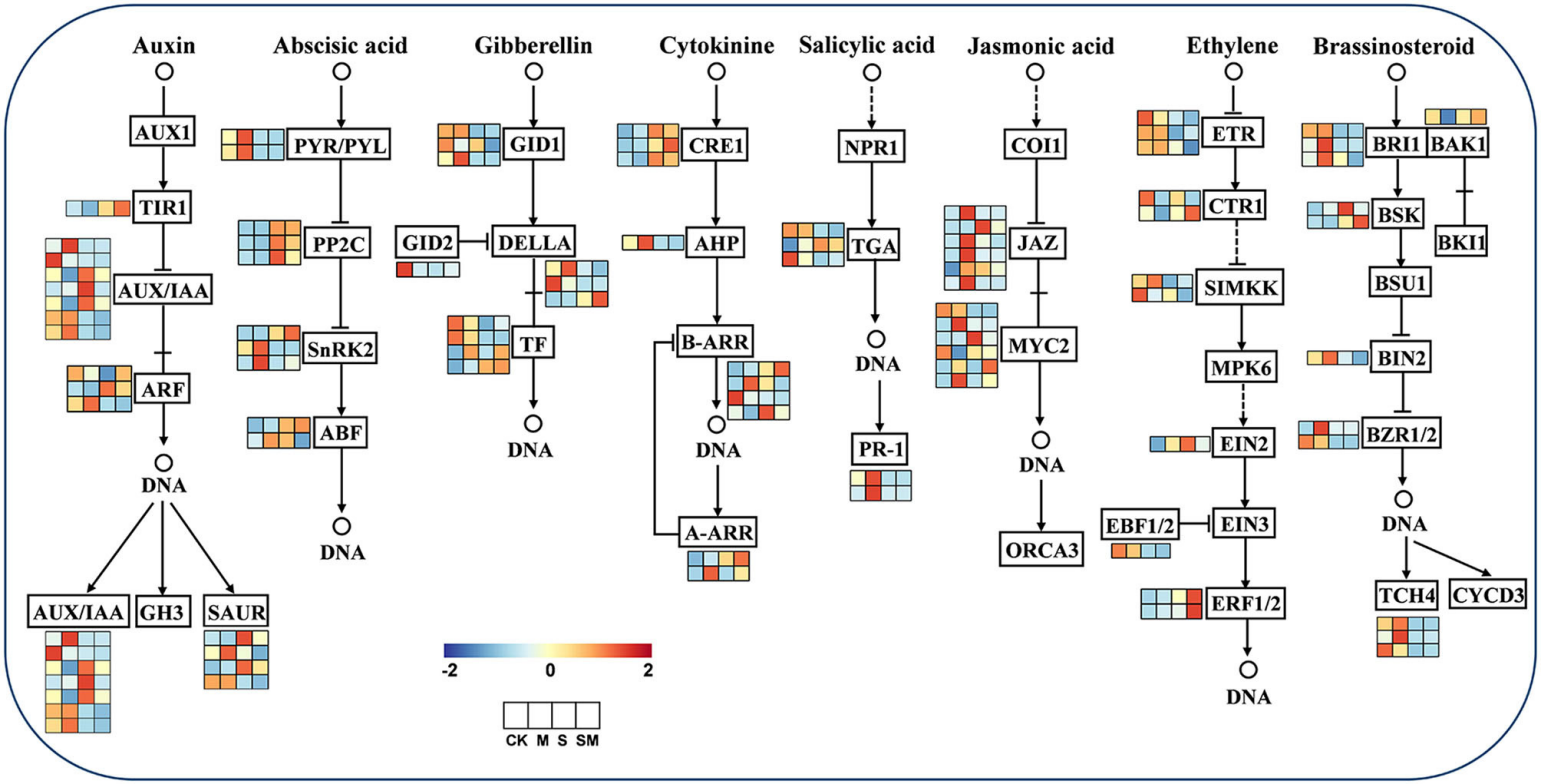
Expression profiles of the plant hormone signal transduction pathway genes in response to MT and salt treatment.

To explore whether melatonin participates in regulating other plant hormones in alfalfa salt tolerance, the expression profiles of the genes related to plant hormone signal transduction were analyzed (Figure 8 and Supplementary Table 10). Furthermore, we predicted the key protein–protein interaction (PPI) network of proteins in the hormone signaling pathway (Supplementary Figure 4).

For the auxin signal, nine genes were differentially expressed in “M *vs*. CK,” two of which were upregulated, genes encoding auxin-induced protein (*AUX28*) and auxin response factor (*ARF19*). In addition, *AUX10, AUX28*, and *IAA6* appear to be central genes in “SM *vs*. S.” Under salt stress, the expression of *IAA27*, SAUR-like auxin–responsive family protein, *IAA8*, and *IAA22D* was induced in “S *vs*. CK,” and transport inhibitor response 1 (*TIR1*) and *ARF3* expression was induced in “SM *vs*. CK.” For the ABA signal, two *PYR/PYLs* (*PYL4* and *PYR1*) were downregulated under salt stress in “S *vs*. CK” and “SM *vs*. CK,” while *PYL9* and protein phosphatase 2C (*PP2C24* and *PP2C51*) were upregulated. In addition, serine/threonine-protein kinase *SRK2A* was upregulated and *PP2C51* was downregulated in “M *vs*. CK.” For the GA signal, the genes encoding F-box proteins (*GID2*), scarecrow-like proteins (*SCL14* and *SCL30*), and phytochrome interacting factor 3 (*PIF3*) were downregulated in “S *vs*. CK,” whereas *SCL9* was upregulated in “S *vs*. CK” and “SM *vs*. CK.” Moreover, the *PIF4* gene was upregulated in “M *vs*. CK” and “SM *vs*. CK,” indicating that it was induced by melatonin. For the CTK signal, CTK receptor histidine kinases (*AHK4*) and response regulator and transcription factor RR-A-type family genes were significantly upregulated in both “S *vs*. CK' and “SM *vs*. CK,” while histidine-containing phosphotransfer proteins (*AHP1*) and two-component response regulators (*ARR12* and *ARR5*) were downregulated. However, melatonin upregulated the expression of *ARR5* in “M *vs*. CK” and “SM *vs*. S.”

For the SA signal, the SA receptor gene *NPR3* and pathogenesis-related protein (*PR1*) were downregulated in “S *vs*. CK” and “SM *vs*. CK,” while the downstream TGA TFs of NPR showed different expression patterns in SA signaling. For the JA signal, three *JAZs* (*TIFY3, TIFY10A* and *TIFY6B*) were upregulated in both “M *vs*. CK” and “S *vs*. CK,” whereas melatonin also induced the expression of *TIFY5A, TIFY10B*, and *TIFY11B* in “M *vs*. CK.” The *MYC2s* showed varied expression levels in JA signaling under salt and melatonin treatments, in which *bHLH18* was upregulated in “S *vs*. CK” and “SM *vs*. CK,” but downregulated in “SM *vs*. S.” For the ETH signal, salt stress inhibited the expression of ethylene receptor 2 (*ETR2*) and MEK map kinase kinase (*SIMKK*) in “S *vs*. CK” and “SM *vs*. CK.” However, the ethylene receptor *EIN4* and *SIMKK* were significantly upregulated in “SM *vs*. S.” Serine/threonine–protein kinase *CTR1* was downregulated in “M *vs*. CK” and “SM *vs*. CK,” whereas it remained unchanged in “S *vs*. CK.” In addition, ethylene-responsive transcription factor 1B (*ERF1B*) was downregulated in all comparisons except “SM *vs*. S.” The EIN3-binding F-box protein 1 (*EBF1*) was downregulated in both “S *vs*. CK' and “SM *vs*. CK.” For the BR signal, brassinazole-resistant 1 (*BZR1*), brassinosteroid LRR receptor kinase (*BRL*), and xyloglucan endotransglucosylase/hydrolase protein 23 (*XTH23*) were downregulated in “S *vs*. CK” and “SM *vs*. CK,” whereas they were upregulated in “M *vs*. CK” comparison. In addition, serine/threonine–protein kinase *BSK3* was upregulated under both salt stress and melatonin treatment.

### Detection of the Phytohormone Contents of Alfalfa Under Salt Stress

**Figure 9:**
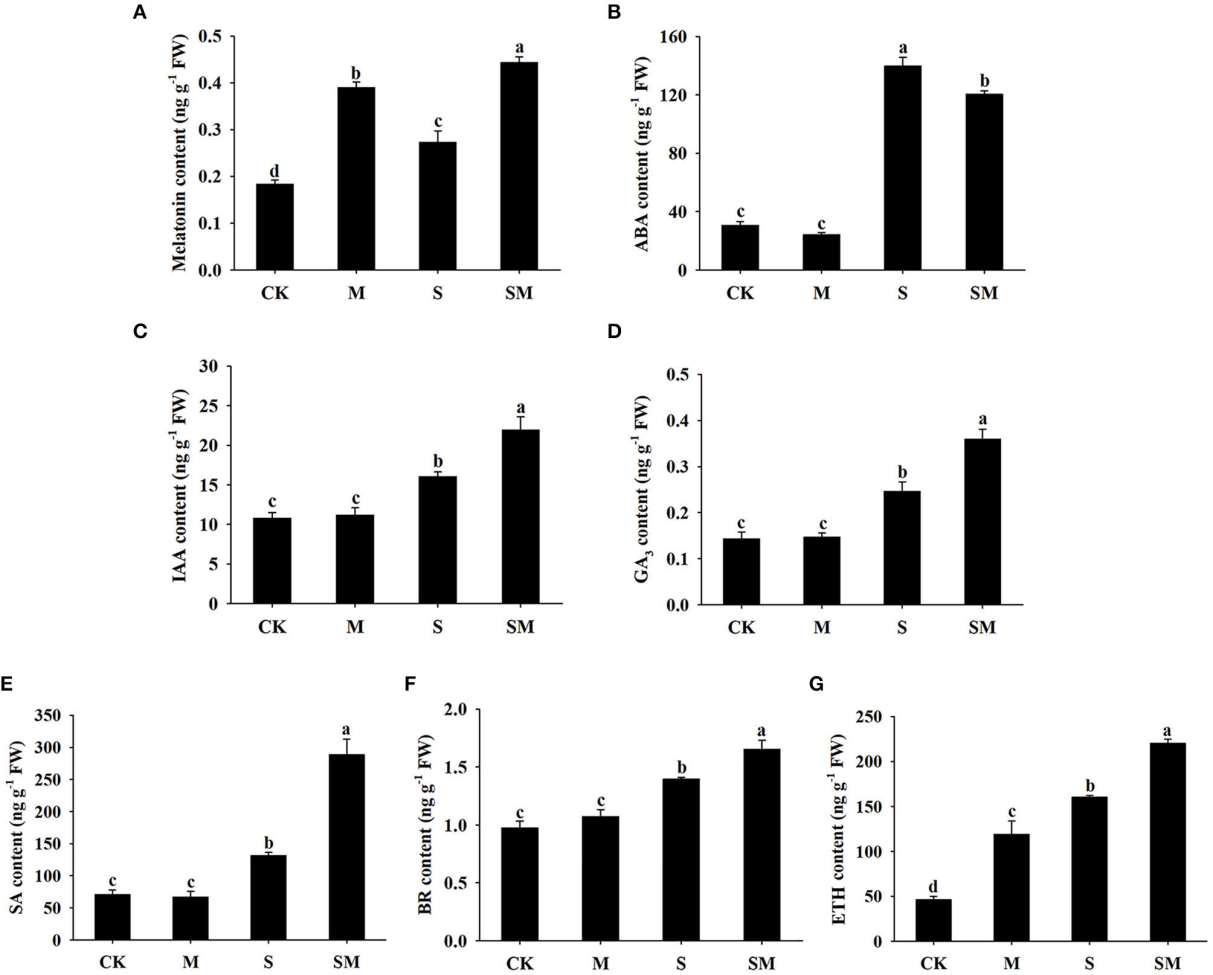
Effect of exogenous melatonin on the endogenous hormone levels of alfalfa seedlings after 15 days of 150-mM NaCl treatment. **(A)** Melatonin content, **(B)** ABA content, **(C)** IAA content, **(D)** GA_3_ content, **(E)** SA content, **(F)** BR content, and **(G)** ETH content. The data are means ± SE (*n* = 3) and different letters are significantly different (*p* < 0.05).

To evaluate the effect of exogenous melatonin on the phytohormone levels of alfalfa, we detected the contents of melatonin, ABA, IAA, GA_3_, SA, BR, and ETH. Herein, we observed that melatonin treatment significantly increased the endogenous melatonin content of alfalfa before and after salt treatment (Figure 9A). As shown in Figure 9B, salt stress significantly increased the endogenous ABA content in comparison with the control plants. However, the application of melatonin decreased the ABA content under salt stress. The contents of IAA, GA_3_, SA, and BR showed similar trends under treatment conditions. Under salt stress conditions, the IAA, GA_3_, SA, and BR contents were remarkably accumulated compared with the control, while melatonin markedly increased the contents in alfalfa (Figures 9C–F). Melatonin-treated plants exhibited obviously higher ETH content than the control plants. Salt stress resulted in a significant increase in the ETH content compared with the control, while melatonin application further enhanced ETH accumulation under salt stress (Figure 9G).

### Validation of DEGs by qRT-PCR

To validate the accuracy and reproducibility of the RNA-seq data, 20 DEGs, including hub genes in starch and sucrose metabolism and plant hormone signal transduction, were randomly selected for qRT-PCR. The expression patterns of all genes were highly consistent with the RNA-seq data (Supplementary Figure 5), which confirms that the DEGs identified in this study are credible.

## Discussion

Salt stress is one of the main factors affecting sustainable agricultural development. Melatonin has been reported to be involved in plant adaptive responses to salt stress in various plants (Yan et al., 2019; Liu et al., 2020; Zhang et al., 2021). Here, we analyzed the key mechanisms by which melatonin enhanced salt tolerance in alfalfa at the physiological and molecular levels. Our results suggest that melatonin could effectively alleviate oxidative damage and ion toxicity by improving photosynthetic capacity, antioxidant defense system, proline content, and the K^+^/Na^+^ ratio. Combined with the changes in starch, sugar, and hormone contents, and the expression of key genes in the transcriptome, our study indicates that melatonin is highly involved in starch and sugar metabolism and plant hormone signal transduction to regulate salt stress.

In the current study, the application of melatonin mitigated the growth inhibition of alfalfa under salt stress, which was reflected in superior plant height, leaf area, and fresh weight (Figures 1A–D). Exogenous melatonin reduces the detrimental effects of salinity on photosynthetic capacity (Chen et al., 2018; Liu et al., 2020). Consistent with these findings, higher Pn and Fv/Fm were observed in melatonin-treated alfalfa under salt stress conditions (Figures 1E,F). Salt stress causes excessive accumulation of ROS, which leads to cell membrane damage and oxidative stress (Miller et al., 2010). Melatonin is known as a broad-spectrum antioxidant that scavenges ROS and increases antioxidant enzyme activity under salt stress (Reiter et al., 2016). Herein, we found that melatonin treatment reduced electrolyte leakage, MDA content, and ROS accumulation under salt stress, while the activities of SOD, POD, GST, and APX were increased (Figure 2). These results suggest that melatonin alleviates oxidative stress mainly by enhancing the activities of antioxidant enzymes in alfalfa plants. Proline acts as an osmolyte and participates in melatonin-regulated salt stress tolerance (Siddiqui et al., 2019). This is consistent with our results, as we found that melatonin led to a remarkable increase in the proline content of alfalfa under salt stress (Figure 3A).

Melatonin, RBOH-mediated ROS, and Ca^2+^ exhibit complex signaling crosstalk to regulate plant responses to salt stress (Wei et al., 2018; Liu et al., 2020). The elevation of intracellular Ca^2+^ induced by salt stress sequentially activates CNGCs, CDPKs, or CAM/CMLs (Srivastava et al., 2013; Gao et al., 2020), while RBOHs can be activated by CDPKs (Drerup et al., 2013; Dubiella et al., 2013). In our study, melatonin activated the expression of *CNGCs* (such as *CNGC1* and *CNGC20*), *CaM/CMLs* (such as *CAM7, CAM11*, and *CAM24*), and *CDPKs* (such as *CDPK1, CDPK11*, and *CDPK28*) in the Ca^2+^ signaling pathway, which in turn downregulated *RBOHs* (*RBOHB* and *RBOHD*) (Supplementary Table 7). A recent study by Liu et al. (2020) revealed that melatonin activated Ca^2+^ signaling to mediate RBOH, which is critical for changes in transcriptional profiles and high- and low-affinity K^+^ transporter activity, thereby improving the salt tolerance of rice. This may be related to the higher K^+^/Na^+^ in melatonin-treated plants under salt stress conditions (Figure 3F).

Sugars act as osmoprotectants to participate in the abiotic stress response in plants (Wang et al., 2016; Yang et al., 2019). Stress-induced starch-to-sugar conversion promotes the accumulation of sugar, which provides osmoprotection and energy supplies for plants (Dong and Beckles, 2019). In sucrose biosynthesis and degradation, SPS and SUS are the key enzymes (Winter and Huber, 2000). Salt reduces the activities of GBSS and AGPase; thus, inhibiting the synthesis of starch (Libalweksler et al., 1994; Chen et al., 2008). BAM1 and AMY3 were also activated by osmotic stress to degrade starch into sugar (Thalmann et al., 2016). However, Yin et al. (2010) reported that salinity increased AGPase activity and enhanced carbohydrate accumulation as starch during the early development stages in tomato. In our study, the contents of sugar and starch in alfalfa increased obviously under salt stress (Figures 3B,C). Interestingly, salt stress upregulated α-glucan phosphorylase, *BAM, AMY2, SS, AGPs, GBSS*, and *SPS2*, while it downregulated *AMY* and *SUS* (Supplementary Table 8). This result indicates that, during a certain stage of salt treatment, the contents of starch and sugar in alfalfa could be simultaneously maintained at high levels to resist stress.

The previous reports have revealed that melatonin could enhance abiotic stress resistance by maintaining a higher accumulation of carbohydrates in bermudagrass (Shi et al., 2015). In our study, melatonin treatment increased the accumulation of starch, which was a major reason for the higher photosynthetic capacity of melatonin-treated plants under salt stress. The sugar and starch content increased in both salt-tolerant and salt-susceptible tomato leaves, but the tolerant genotype had higher starch accumulation (Balibrea et al., 2000). Plants convert a portion of sugars to starch to minimize the physiological damage of excess sugar in the source leaves (Dong and Beckles, 2019). According to our data, the genes *EDGL6, EGLC13, EGLC14, GBSS2, BAM*, and *FRK7* were upregulated in “M *vs*. CK,” while six *SUSs* were downregulated (Figure 6B and Supplementary Table 8). The melatonin-induced expression changes of most genes were more conducive to the accumulation of starch, thus reducing the content of sucrose. Higher starch is proposed to increase starch statoliths and gravitropic response, and to direct root growth for the acquisition of nutrients, minerals, or water under salt stress (Baldwin et al., 2013; Thitisaksakul et al., 2017). The isoforms of BGLU located to various organelles catalyze the single-step hydrolysis of ABA-glucose ester to produce ABA, and they become activated upon stress (Han et al., 2020). Wang et al. (2021) have revealed that melatonin regulates *BGLU18* mediated ABA-glucose ester hydrolysis to modulate ABA homeostasis and abiotic stress responses. We found that *BGLU12* and *BGLU46* were downregulated in the “SM *vs*. S” comparison, indicating that they may be associated with the ABA signaling pathway. Moreover, a high Na^+^ concentration stimulates the accumulation of starch in common reed (*Phragmites australis*), which leads to the deposition of Na^+^ in starch granules, thus achieving “ion-trapping” to maintain cellular osmotic balance (Kanai et al., 2007). Melatonin treatment may be beneficial to this process, which needs to be further verified. Taken together, these results suggest that salt stress activates the starch and sucrose metabolism to maintain a high and balanced level; moreover, the application of melatonin was beneficial to the maintenance of the starch-sucrose ratio to improve the salt tolerance of alfalfa.

Transcription factors have been reported to play important roles in salt stress responses, which uniquely regulate and modify different stress-responsive genes. Many TFs have been identified to be involved in melatonin-mediated stress tolerance. Most of these were stress-related TFs, including MYBs, WRKYs, NACs, and zinc finger-related TFs (Zhang et al., 2014; Zhan et al., 2021). In this study, various TFs were significantly differentially expressed in different comparisons, among which C3H, MYB, and ERF were the three most abundant families (Figure 7). The largest proportion of the C3H family contained 69 and 55 DEGs in “M *vs*. CK” and “SM *vs*. S,” respectively, suggesting that these TFs participated in salt resistance regulated by melatonin. In tomato, *DREB1*α and *IAA3* are key downstream transcription factors of melatonin-induced sodic alkaline stress tolerance at the genetic level (Yan et al., 2019). These results indicate that TFs might contribute to enhancing the salt tolerance of melatonin-treated alfalfa.

It has been shown that phytohormones, act as secondary messengers, participate in stress sensing and signal transduction through antagonistic or synergistic action, and activate downstream transcription factors to regulate stress responses (Long and Benfey, 2006; Yan et al., 2019). In this study, we focused on the major hormone signaling pathways under salt and melatonin treatments and their possible crosstalk. Salt stress induces changes in endogenous melatonin levels in different plant species (Li et al., 2019c; Zhang et al., 2021). It has been reported that *COMT* and *SNAT* are pivotal genes for melatonin biosynthesis (Arnao and Hernandez-Ruiz, 2014). Herein, we observed a significant increase in endogenous melatonin levels in both the control and salt-treated plants after melatonin treatment (Figure 9A). In addition, the expression of *COMT* was upregulated under salt stress, and its extent of upregulation in “SM *vs*. CK” was higher than that in “S *vs*. CK” (Supplementary Figure 5). The results suggest that melatonin is closely related to the salt tolerance of alfalfa, and consistent results were found in cotton and tomato (Yan et al., 2019; Zhang et al., 2021).

Auxins play key roles in regulating plant growth and development, and they can govern the growth response of plants to stress (Eyidogan et al., 2012). Auxin-responsive genes have been separated into three major classes: Aux/IAA, GH3 and SAUR (Hagen and Guilfoyle, 2002). There are at least two auxin receptors reported in the literature: nuclear TIR1/AFB Aux/IAA coreceptor and auxin-binding protein 1 (ABP1) (Grones and Friml, 2015). Stress pathways interact with the auxin gene regulatory network through the transcription of *Aux/IAA* genes (Shani et al., 2017). In this study, melatonin suppressed almost all *Aux/IAA* and *SAUR* genes under salt stress, but induced *TIR1* and *ARF3* expression (Supplementary Table 10). Chen et al. (2017) reported that overexpression of *CsTIR* enhanced salt tolerance in transgenic Arabidopsis. It has been reported that melatonin treatment induces a slight increase in endogenous IAA in *Brassica juncea* and tomato (Chen et al., 2009; Wen et al., 2016). Our results showed that endogenous IAA content in alfalfa was significantly increased after melatonin application under salt stress (Figure 9C). The results indicate that melatonin may alleviate salt stress by modulating the expression of auxin coreceptors and response genes and increasing auxin content.

Abscisic acid has been proposed as a stress hormone because it acts as an important internal signal mediating plant responses to stress, and stress tends to induce ABA synthesis (Fahad et al., 2015). In the ABA biosynthetic pathway, when PYR/PYL/RCAR binds to ABA, the complex interacts with PP2C to reduce the inhibition of SNF1-related kinases (SnRKs), thus activating their downstream transcription factors (Yang et al., 2019). ABA oxidase (AAO) and 9-cis-epoxycarotenoid dioxygenases (NCED) are key enzymes in the ABA biosynthetic pathway (Mcadam et al., 2015). In this study, salt stress induced *PYL9, PP2Cs, AAO1*, and *NCEDs* expression and ABA accumulation, while repressing *PYL4* and SnRK2s expression. However, melatonin treatment increased SRK2A expression in the “M *vs*. CK” comparison (Supplementary Table 10). Di et al. (2018) reported that *BnPYL9-1* and *BnPYL9-2* expression was inhibited by salinity stress, which may be due to negative feedback regulation caused by high ABA accumulation. Also, SnRK2s are key regulators governing plant adaptive responses to osmotic stresses. Soma et al. (2020) reported that subclass I SnRK2s (SRKs) are rapidly activated by osmotic stress prior to ABA accumulation, implying that SRK2A may not be activated by ABA. Melatonin treatment upregulated the expression of ABA catabolism gene *CYP707A1* and reduced ABA accumulation under salt stress. This result is consistent with that in tomato reported by Hu et al. (2021). In addition, melatonin treatment resulted in downregulated expression of *PP2C51*. The previous studies have revealed that *PYL4* and *PP2Cs* are considered to be central genes that interact with ERF and GA metabolic genes, and that melatonin plays a regulatory role upstream of the ABA signaling pathway (Wang et al., 2021). In this study, we speculate that PP2Cs interacted with ETH and IAA metabolic genes in the “S *vs*. CK” and “SM *vs*. CK” comparisons (Supplementary Figure 4). These results suggest that melatonin might enhance the salt tolerance of alfalfa by mediating *PP2C51* and *CYP707A1* expression and crosstalk with ETH and IAA signals.

As a key messenger and integrator of intrinsic growth responses, GA is also involved in regulating plant responses to salt stress. Gibberellic acid signaling is mediated by its receptor GID1, the repressor DELLA, and the F-box protein GID2; thus, triggering downstream responses (Kohli et al., 2013). The repressor, DELLA, controls the GA signaling pathway by antagonizing the GA-positive regulator *SCL3* promoter sequence and blocks PIF transcriptional regulation activity to orchestrate GA homeostasis (Hirano et al., 2008). The findings of our study showed that salt stress inhibited *SCLs* and *PIF3* expression, while melatonin treatment resulted in upregulation of *PIF4* and *SCL13* to regulate GA signaling. Zhang et al. (2014) reported that melatonin increased GA content by promoting the expression of the GA synthesis genes *GA20ox* and *GA3ox* under salt stress conditions. Similarly, our results also showed that *GA2ox2* was upregulated after melatonin application. The repressor, DELLAs, orchestrate the crosstalk between GA and other plant hormones, such as ABA and ETH, to participate in the salt stress response (Achard et al., 2006). In our study, *PIFs* and *SCLs* were hub genes in the GA signaling pathway, and *SCLs* interacted with the genes involved in ABA and SA signal transduction to alleviate salt stress in alfalfa.

Ethylene is considered a stress hormone required in various abiotic stress responses in plants. Salt stress promotes ethylene production in plants by regulating the activities of 1-aminocyclopropane-1-carboxylate (ACC) synthase (ACS) and ACC oxidase (ACO) (Achard et al., 2006; Dong et al., 2011). Melatonin enhances salt tolerance by promoting *MYB108A*-mediated ethylene biosynthesis in grapevines (Xu et al., 2019). In our study, salt stress and melatonin treatment increased the ETH content in alfalfa (Figure 9G), indicating that melatonin could defend against salt stress by regulating ethylene levels. The ERFs are the major downstream regulatory factors of the ETH signaling pathway in stress responses. Although *ERF1B* was identified as a positive regulator of salt stress tolerance, its expression was repressed under salt treatment in *Chrysanthemum* (Gao et al., 2015). Similarly, *ERF1B* was downregulated by melatonin under salt stress (Supplementary Table 10). The regulatory effect of melatonin on ethylene synthesis may be related to complex hormone signal crosstalk (Xu et al., 2019). Ethylene-responsive transcription factor can combine with ABA to affect stomatal opening under drought stress and through the combination of ERFs, GA, and CIPK under low-oxygen stress to play a specific role (Kohli et al., 2013). In this study, *ERF1B* was associated with other hormonal signaling components, such as *ARF2A, TIFY10A*, and *MYC2*, indicating that hormonal signals regulate salt stress through a crosstalk relationship.

Salicylic acid participates in defense responses to a variety of environmental stresses including salinity. Salicylic acid signaling leads to the activation of the NPR1, which is thought to be recruited to numerous downstream PRs by transcription factors such as TGAs (Jin et al., 2018). Li et al. (2019a) reported that overexpression of *TGA17* enhanced the salt tolerance of soybean. Our data showed that melatonin induced the upregulation of *TGA3* and *TGA7* under salt stress. Moreover, cytokinin-activated transcription factor ARR2 in Arabidopsis promotes plant immunity through a TGA3/NPR1-dependent salicylic acid signaling pathway (Choi et al., 2010). Here, the hub gene *TGA* was associated with the *ETR1, SCL14*, and *MYC2* genes in the ETH, GA and JA signaling pathways, which suggested that crosstalk occurred between these phytohormones. Taken together, these results reveal the crosstalk relationship of plant hormones and provide new insights into the involvement of hormone signals in salt stress responses.

Jasmonic acid is known to play major roles in mediating the plant defense response against salt stress. The transcription factor MYC2 and repressor protein JAZ play crucial roles in the JA response under stress conditions (Verma et al., 2016). Under JA-stimulated conditions, JA-Ile (bioactive JA) binds to its receptor, an F-box protein CORONATINE INSENSITIVE1 (COI1), and leads to 26S proteasome-mediated degradation of JAZ, thereby allowing for MYC2 to upregulate the expression level of JA target genes (Chini et al., 2007). In this study, salt stress and melatonin treatment induced the expression of *TIFY3, TIFY10A, TIFY6B*, and *MYC2* (*bHLH18*), indicating that they might play important regulatory roles against salt stress in alfalfa (Supplementary Table 10). This is consistent with the regulation of JA signaling by melatonin in loquat under drought stress (Wang et al., 2021).

The previous studies have demonstrated that melatonin and BRs synergistically regulate plant morphogenesis (Hwang and Back, 2018; Fu et al., 2022). However, there are still divergent views on the crosstalk of melatonin and BR signaling in abiotic stress (Hwang and Back, 2019). In this study, melatonin treatment increased the BR content under salt stress (Figure 9F). In addition, melatonin induced the transcription levels of genes related to BR signaling including *BZR1, BRL, BSK3*, and *XTH23* in alfalfa (Supplementary Table 10), indicating that melatonin and BR function together in response to salt stress. These findings are consistent with the study of Fu et al. (2022), who reported that melatonin-induced cold and drought tolerance is regulated by BR in perennial ryegrass. BR-mediated stress tolerance in Arabidopsis was associated with the ABA, SA, and ETH pathways (Divi et al., 2010). In this study, the BR LRR receptor kinase CURL3 interacted with EIN3, NPR1, IAA6, PP2C51, and GID2 under salt stress, indicating that there may be a crosstalk relationship between BR and ETH, SA, IAA, ABA, and GA signaling (Supplementary Figure 4).

**Figure 10:**
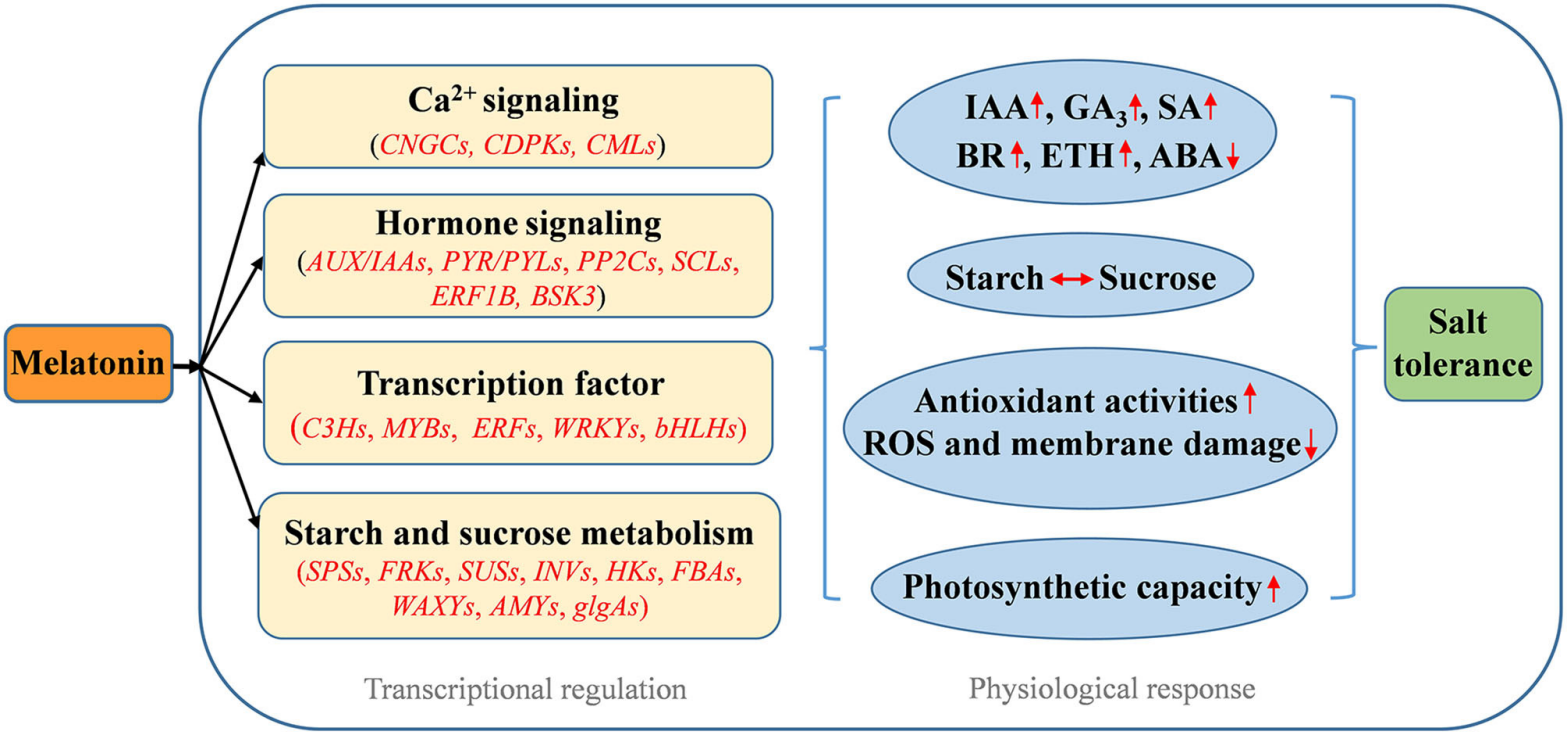
Schematic model for melatonin-induced salt stress tolerance in alfalfa.

In conclusion, this study provides new insight into the protective roles of melatonin against salt stress in alfalfa. Based on physiochemical and transcriptomic data, a schematic model for the regulation of the alfalfa salt stress response by melatonin is presented in Figure 10. Melatonin alleviated salt stress by increasing starch accumulation to maintain high photosynthetic capacity and enhancing the antioxidant defense system to scavenge excess ROS accumulation. Melatonin improved the salt stress tolerance of alfalfa mainly by mediating the profiles of the genes involved in Ca^2+^ signaling and starch and sucrose metabolism. Melatonin participated in mediating other plant hormone signal transduction pathways and affected endogenous hormone levels by regulating related genes under salt stress. Moreover, the crosstalk relationship among important phytohormone signaling pathways in alfalfa under salt stress is evidenced. Our results lay a foundation for further research on the molecular mechanisms of the melatonin-induced salt stress response.

## Data Availability Statement

The datasets presented in this study can be found in online repositories. The names of the repository/repositories and accession number(s) can be found below: NCBI Gene Expression Omnibus (GEO) database, accession no: GSE199945.

## Author Contributions

SL and BF designed the experiments and wrote the manuscript. SL, YW, XG, and JL performed the experiment. YW and XG analyzed the data and prepared the figures. JL and BF provided ideas and revised the manuscript. All authors contributed to the article and approved the submitted version.

## Funding

This work was supported by the National Natural Science Foundation of China (32101426), Ningxia Natural Science Foundation (2022AAC03124), and Ningxia Hui Autonomous Region Agricultural Breeding Special Project (2019NYYZ0403).

## Conflict of Interest

The authors declare that the research was conducted in the absence of any commercial or financial relationships that could be construed as a potential conflict of interest.

## Publisher's Note

All claims expressed in this article are solely those of the authors and do not necessarily represent those of their affiliated organizations, or those of the publisher, the editors and the reviewers. Any product that may be evaluated in this article, or claim that may be made by its manufacturer, is not guaranteed or endorsed by the publisher.
