# Supplementary material for: Comparative Physiological and Transcriptome Analysis Reveal the Molecular Mechanism of Melatonin in Regulating Salt Tolerance in Alfalfa (Medicago sativa L.)
Source: Front Plant Sci. 2022 Jul 13;13:919177. doi: 10.3389/fpls.2022.919177 (PMC9326453; doi:10.3389/fpls.2022.919177)
Supplement: Supplementary file 1 [file Data_Sheet_1.ZIP › Supplementary Material/Supplementary Figure 1. Root phenotype and melatonin treatment concentration selection.pdf]

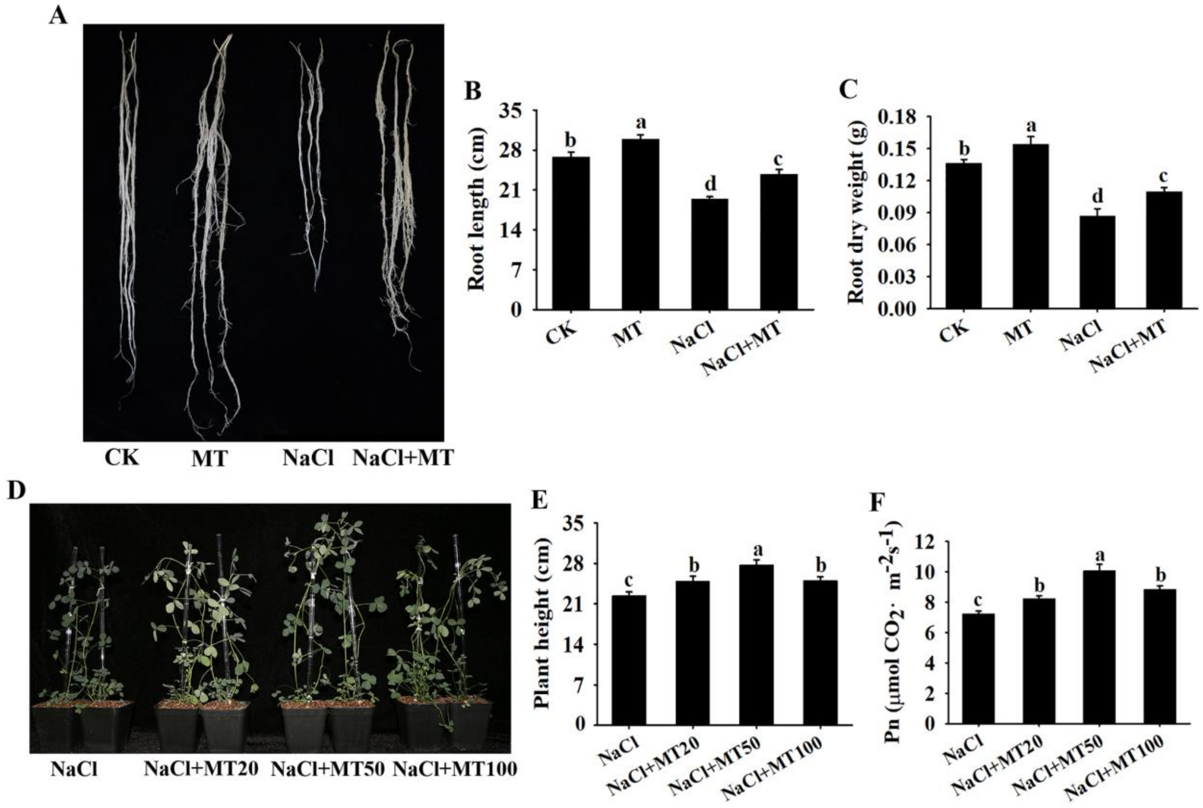


Supplementary Figure 1. Effects of 10 μM melatonin treatment on root phenotype traits (A), root length (B) and root dry weight (C) of alfalfa under salt stress in hydroponic experiments. Effects of different concentrations of melatonin (0, 20, 50, and 100 μM) treatment on phenotype traits (D), plant height (E) and net photosynthetic rate (Pn) (F) of alfalfa under salt stress in soil culture. Data are means ± SE (n=6) and different letters are significantly different ($P < 0.05$).
