# Supplementary material for: Comparative Physiological and Transcriptome Analysis Reveal the Molecular Mechanism of Melatonin in Regulating Salt Tolerance in Alfalfa (Medicago sativa L.)
Source: Front Plant Sci. 2022 Jul 13;13:919177. doi: 10.3389/fpls.2022.919177 (PMC9326453; doi:10.3389/fpls.2022.919177)
Supplement: Supplementary file 1 [file Data_Sheet_1.ZIP › Supplementary Material/Supplementary Figure 3. GO term.pdf]

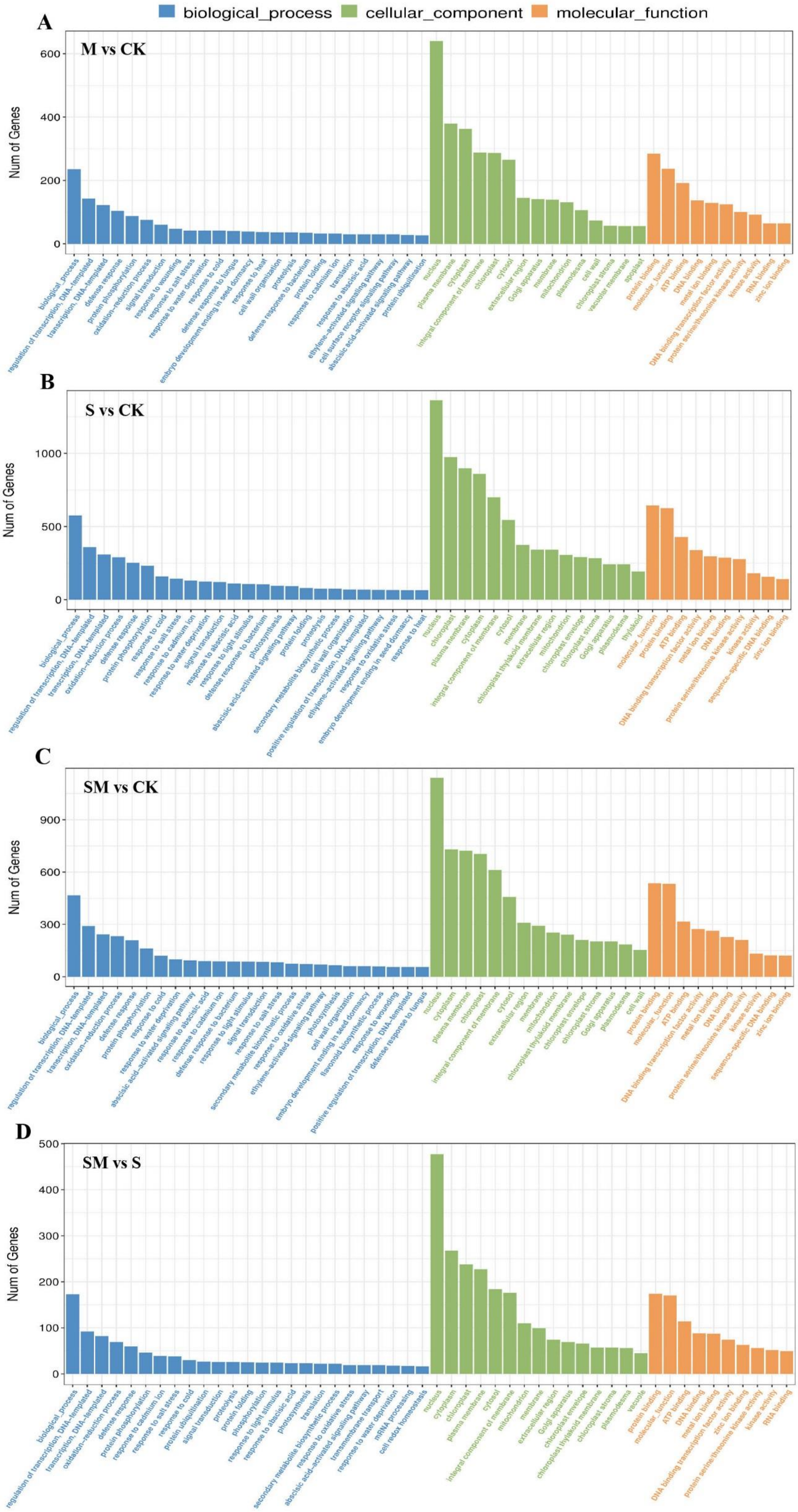

biological_process
cellular_component
molecular_function
A
M vs CK
Num of Genes
0
200
400
600
B
S vs CK
Num of Genes
0
500
1000
C
SM vs CK
Num of Genes
0
300
600
900
D
SM vs S
Num of Genes
0
100
200
300
400
500

Supplementary Figure 3. GO enrichment analysis of DEGs in different comparisons. (A) Classification of the enriched GO terms in ‘M vs CK’. (B) Classification of the enriched GO terms in ‘S vs CK’. (C) Classification of the enriched GO terms in ‘SM vs CK’. (D) Classification of the enriched GO terms in ‘SM vs S’.
