# Supplementary material for: Comparative Physiological and Transcriptome Analysis Reveal the Molecular Mechanism of Melatonin in Regulating Salt Tolerance in Alfalfa (Medicago sativa L.)
Source: Front Plant Sci. 2022 Jul 13;13:919177. doi: 10.3389/fpls.2022.919177 (PMC9326453; doi:10.3389/fpls.2022.919177)
Supplement: Supplementary file 1 [file Data_Sheet_1.ZIP › Supplementary Material/Supplementary Figure 4. PPI network.pdf]

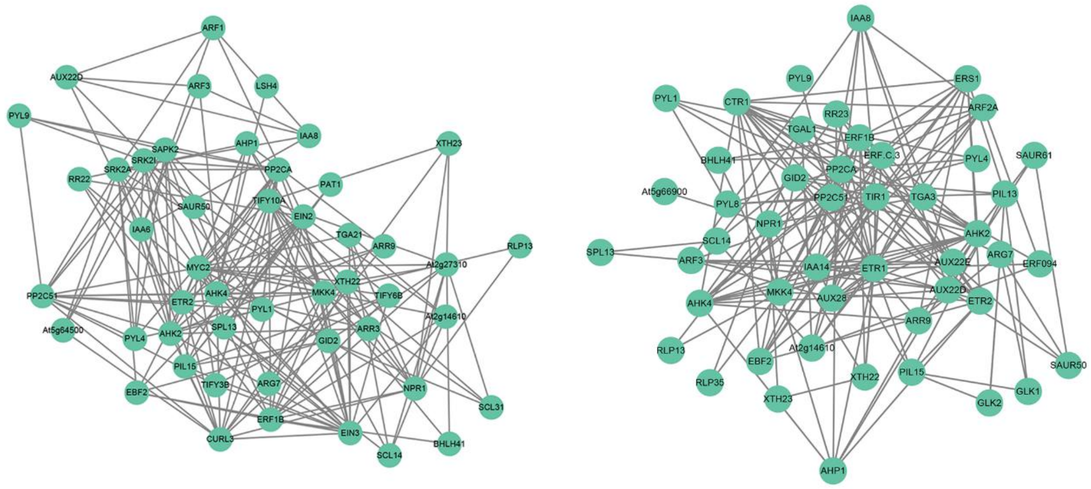

S vs CK **SM vs CK**

Supplementary Figure 4. PPI network for hormone signal transduction genes in 'S vs CK' and 'SM vs CK'.
