# Supplementary figures and images for: Comparative Physiological and Transcriptome Analysis Reveal the Molecular Mechanism of Melatonin in Regulating Salt Tolerance in Alfalfa (Medicago sativa L.)
Source: Front Plant Sci. 2022 Jul 13;13:919177. doi: 10.3389/fpls.2022.919177 (PMC9326453; doi:10.3389/fpls.2022.919177)

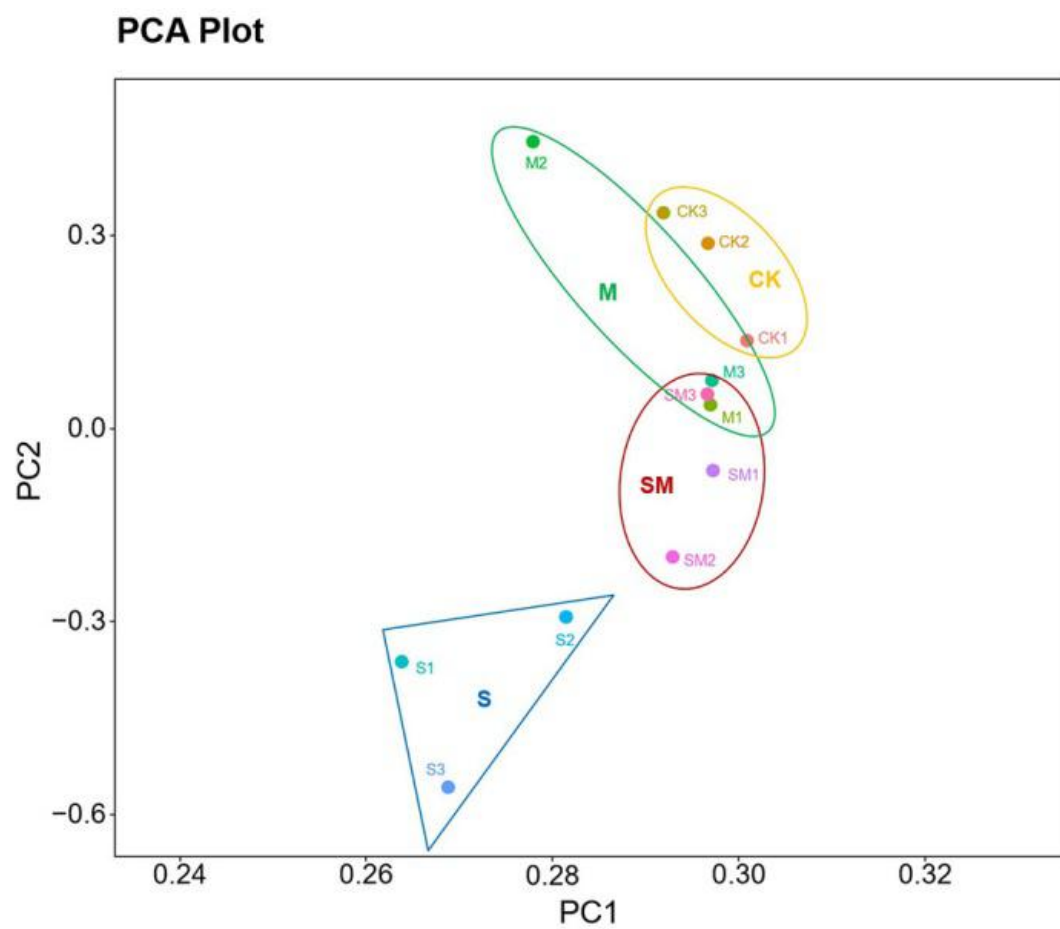

Supplementary Figure 2. PCA analysis

Supplement: Supplementary file 1 [file Data_Sheet_1.ZIP › Supplementary Material/Supplementary Figure 2. PCA analysis.pdf]

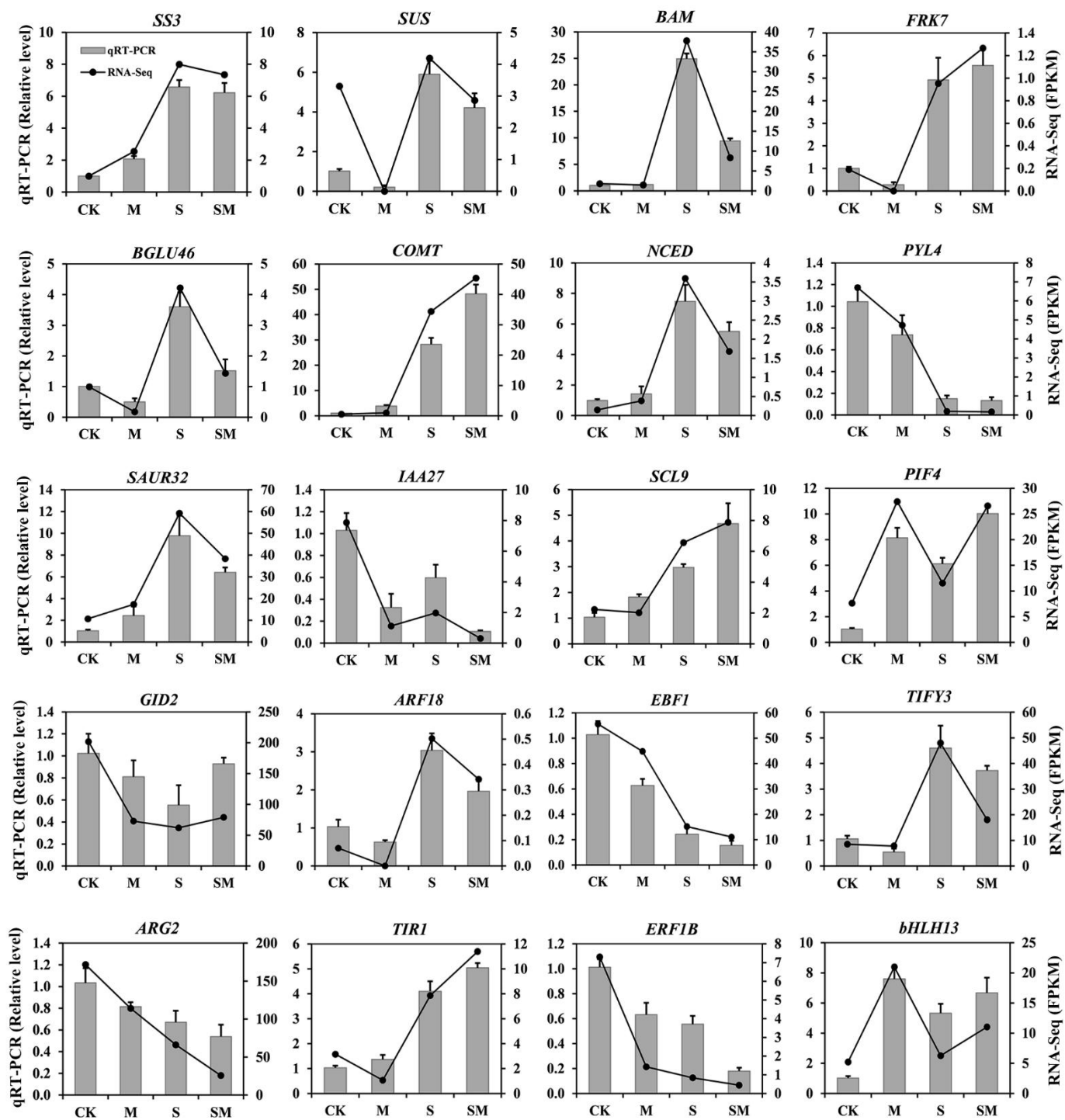

Supplementary Figure 5. qRT-PCR validation of the expression level of selected genes.

Supplement: Supplementary file 1 [file Data_Sheet_1.ZIP › Supplementary Material/Supplementary Figure 5. qRT-PCR validation.pdf]
